# Tracking Public Beliefs About Anthropogenic Climate Change

**DOI:** 10.1371/journal.pone.0138208

**Published:** 2015-09-30

**Authors:** Lawrence C. Hamilton, Joel Hartter, Mary Lemcke-Stampone, David W. Moore, Thomas G. Safford

**Affiliations:** 1 University of New Hampshire, Durham, New Hampshire, United States of America; 2 Carsey School of Public Policy, University of New Hampshire, Durham, New Hampshire, United States of America; 3 Environmental Studies Program, University of Colorado, Boulder, Colorado, United States of America; 4 Geography Department, Durham, New Hampshire, United States of America; Centro de Investigacion Cientifica y Educacion Superior de Ensenada, MEXICO

## Abstract

A simple question about climate change, with one choice designed to match consensus statements by scientists, was asked on 35 US nationwide, single-state or regional surveys from 2010 to 2015. Analysis of these data (over 28,000 interviews) yields robust and exceptionally well replicated findings on public beliefs about anthropogenic climate change, including regional variations, change over time, demographic bases, and the interacting effects of respondent education and political views. We find that more than half of the US public accepts the scientific consensus that climate change is happening now, caused mainly by human activities. A sizable, politically opposite minority (about 30 to 40%) concede the fact of climate change, but believe it has mainly natural causes. Few (about 10 to 15%) say they believe climate is not changing, or express no opinion. The overall proportions appear relatively stable nationwide, but exhibit place-to-place variations. Detailed analysis of 21 consecutive surveys within one fairly representative state (New Hampshire) finds a mild but statistically significant rise in agreement with the scientific consensus over 2010–2015. Effects from daily temperature are detectable but minor. Hurricane Sandy, which brushed New Hampshire but caused no disaster there, shows no lasting impact on that state’s time series—suggesting that non-immediate weather disasters have limited effects. In all datasets political orientation dominates among individual-level predictors of climate beliefs, moderating the otherwise positive effects from education. Acceptance of anthropogenic climate change rises with education among Democrats and Independents, but not so among Republicans. The continuing series of surveys provides a baseline for tracking how future scientific, political, socioeconomic or climate developments impact public acceptance of the scientific consensus.

## Introduction

“Human activities are changing Earth’s climate,” reads the opening sentence of the American Geophysical Union’s position statement on climate change [1]. The same point is central to statements by other science organizations, broad interdisciplinary reviews [2], direct surveys of scientists [3][4], and literature reviews [5][6]. No major science organization takes a contrary position that human activities are not changing the Earth’s climate [7].

While the scientific consensus has strengthened, public opinion remains seriously divided, without a clear trend [8][9]. Repeated surveys report annual-scale variations possibly related to developments such as release of the 2007 IPCC report, the 2008 economic crisis, “climategate” attacks on scientists in 2009, or a snowy northeastern US winter in 2011 [10][11]. Decadal-scale surveys provide essential perspective, but must employ questions with wording that has changed over the years, or else was frozen at a time when the discourse was different. Whereas recent scientific statements emphasize the term “climate change,” referencing regional differences and shifts in precipitation, storms or extreme events, the legacy survey questions often ask about “global warming” instead. Non-scientists sometimes misinterpret this term to mean that every place should be constantly warming, which seems easily refuted by pointing out a place that is cooling. Moreover, there has been publicity about a “pause” or slowdown in the rate of global air temperature rise, leading to unscientific claims that global warming had stopped [12]. The term “global warming” by itself apparently can elicit more conservative opposition than the term “climate change” on surveys [13]. A potentially greater problem with wording is that some of the longest-running survey questions do not specify human causation, which today (rather than the mere fact of change) forms the main point of public contention [14]. The reality of climate change has been publicly acknowledged even by political leaders who dismiss human causation as a hoax [15]. These complications in public discourse make it harder to interpret responses to survey questions designed long ago.

To unambiguously track public acceptance of the scientific consensus, in 2010 we started asking a question with three response choices:

Which of the following three statements do you personally believe?

Climate change is happening now, caused mainly by human activities.
Climate change is happening now, but caused mainly by natural forces.
Climate change is NOT happening now.

Respondents can also say they don’t know, or decline to answer. Our question is present-tense and neutrally worded, with no mention of policies or future consequences. One response corresponds to the central point of scientific consensus statements, while others present the main logical alternatives. Although some scientists might argue that “belief” is the wrong term for their conclusions, it makes more sense with regard to acceptance by the general public. Trained telephone or face-to-face interviewers read the response choices in rotating order to avoid possible bias. From 2010 to 2015 over 28,000 people answered this question on 35 random-sample surveys, including the benchmark General Social Survey and a unique statewide time series.

Below we synthesize data from all of these surveys, analyzing them in a common multivariate framework. Logistic regression quantifies the effects of respondent age, gender, education and political orientation. This broad replication establishes a set of robust and consistent results. Regional surveys reflect the scale of place-to-place variation in climate-change beliefs, while the single-state time series shows temporal variation, permitting tests for the influence of daily weather, seasons and trends.

## Data

Three US nationwide surveys, 11 surveys in selected, often rural US regions, and a series of 21 surveys in the state of New Hampshire comprise the data for this paper. Individual surveys, which include questions on many topics besides climate, have been introduced in previous papers. Here we undertake the first synthesis bringing all of them together, and analyzing responses to the common climate-change question.

### General Social Survey (GSS 2012, 1,295 interviews)

The climate beliefs question was asked in face-to-face interviews for a panel subset of this representative US survey (variable *clmtchng* in GSS terminology) [16]. The National Opinion Research Center (NORC) at the University of Chicago, supported by the National Science Foundation, conducts the GSS and publishes its data as a resource for research. Intensive sampling and diagnostic efforts make GSS a benchmark for representativeness among US surveys. The 2012 response rate is given as 71%. Our analysis applies probability weights (variable *wtssall*) calculated by NORC.

### National Community and Environment in Rural America Survey (NCERA 2011, 2,006 interviews)

Climate belief and knowledge questions were carried on this representative 50-state telephone survey conducted in summer 2011 [14]. NCERA was developed by researchers at the Carsey School of Public Policy, with sampling and interviewing done by the University of New Hampshire (UNH) Survey Center. The response rate was 31%, as calculated by the American Association for Public Opinion Research (AAPOR) definition 4 [17]. Probability weights (named *ncerawt* in S1 Dataset attached; see S1 File for a complete list of variables) that take account of household size, age-sex-race distributions by region, and metropolitan/nonmetropolitan composition are applied with relatively minor effects.

### iMediaEthics Poll on Climate Change (IME 2014, 1,002 interviews)

Princeton Survey Research Associates International conducted this landline and cell phone survey with a nationally representative sample of adults living in the continental United States. Interviews were done in English and Spanish by Princeton Data Source from July 17–20, 2014. Probability weights (variable *wt2* in S2 Dataset attached) correct for known demographic discrepancies. Wording of the climate change question on other surveys described here is identical, but the context and wording for the iMediaEthics survey are slightly different, as given in the documentation file attached (S3 File).

### New Hampshire Granite State Poll (GSP 2010–2015, 11,548 interviews)

The Granite State Poll conducts telephone interviews with independent random samples of about 500 New Hampshire residents four times each year. Our core climate question has been carried on 21 surveys to date, from April 2010 through May 2015. Sampling and interviews for the GSP are done by the UNH Survey Center, with response rates averaging 25% (AAPOR 2006 definition 4). Probability weights (variable *censuswt2*) provide adjustments for minor design and sampling bias. The S3 Dataset attached contains the climate-change responses from all of the New Hampshire, CERA/CAFOR and other surveys described in this paper, a total of 28,962 individual interviews.

### Community and Environment in Rural America and Communities and Forests in Oregon (CERA 2010–2012 and CAFOR 2011, 2014, 13,111 interviews)

These telephone surveys, done by the UNH Survey Center under direction of Carsey School researchers, employ sampling, interviewing and weighting methods similar to those of NCERA. They target small clusters of counties, many of them nonmetropolitan. The locations are diverse but selected non-randomly for different projects. The CERA and CAFOR surveys used here involve regions in Appalachia, the Columbia River, Gulf Coast Florida, Gulf Coast Louisiana, northern New England, eastern Oregon, the Olympic Peninsula, Puget Sound, and southeast Alaska. Table 1 lists the counties, dates and number of interviews comprising each of these CERA/CAFOR surveys. Citations to many papers describing individual studies are given in [18][19][20]. Response rates for individual surveys (AAPOR 2006 definition 4) range from 18 to 48%, with a mean of 31. For all analyses here we adopt the original CERA or CAFOR weighting schemes, which take into account household size, county adult population and age-sex or age-sex-race distributions.

**Table 1:** Community and Environment in Rural America (CERA) and Communities and Forests in Oregon (CAFOR) surveys that carried the climate-beliefs question. Conducted by Carsey School of Public Policy (formerly Carsey Institute) researchers over 2010 to 2014.[18][19][20] N denotes the number of interviews.

*Appalachia (CERA)*
November 2010–January 2011: Harlan and Lechter Counties in coal country of Kentucky (n = 1,020)
*Blue Mountain (CAFOR)*
August–October 2014: Baker, Crook, Grant, Umatilla, Union, Wallowa and Wheeler Counties, Oregon (n = 1,752)
*Columbia River (CERA)*
January–February 2011: Clatsop County, Oregon and Pacific County, Washington (n = 1,023)
*Gulf Coast Florida (CERA)*
August–September 2010: Bay, Franklin and Gulf Counties along the eastern Gulf Coast of Florida (n = 1,005)
*Gulf Coast Louisiana (CERA)*
Late July–September 2010): Plaquemines and Terrebonne Parishes in coastal Louisiana (n = 1,017)
*Ketchikan*, *Alaska (CERA)*
June–August 2010: Ketchikan Gateway Borough and Prince of Wales Census Area in Southeast Alaska (n = 509)
*North Country (CERA)*
June 2010: Coos County, New Hampshire; Essex County, Vermont; and Oxford County, Maine are adjacent in northern New England (n = 1,852)
*Northeast Oregon (CAFOR)*
September–October 2011: Baker, Union and Wallowa Counties in northeast Oregon (n = 1,585)
*Olympic Peninsula (CERA)*
October–November 2010: Clallam and Grays Harbor Counties, on Washington’s Olympic Peninsula (n = 1,013)
*Puget Sound (CERA)*
January–February 2012: King, Kitsap, Mason and Pierce Counties, in the Puget Sound area of Washington (n = 1,302)
*Southeast Alaska (CERA)*
November–December 2010, with a small number of interviews in February 2011: Haines, Juneau, Sitka, Skagway, Wrangell and Yakutat Boroughs, along with the Hoonah-Angoon and Petersburg Census Areas, all in Southeast Alaska (n = 1,033)

The privacy and interests of subjects interviewed for these surveys are protected through protocols approved by Institutional Review Boards at NORC (for GSS) or UNH (for NCERA, GSP, CERA and CAFOR). All data are recorded, analyzed and presented anonymously, as specified for these protocols.

## Methods

**Fig 1:**
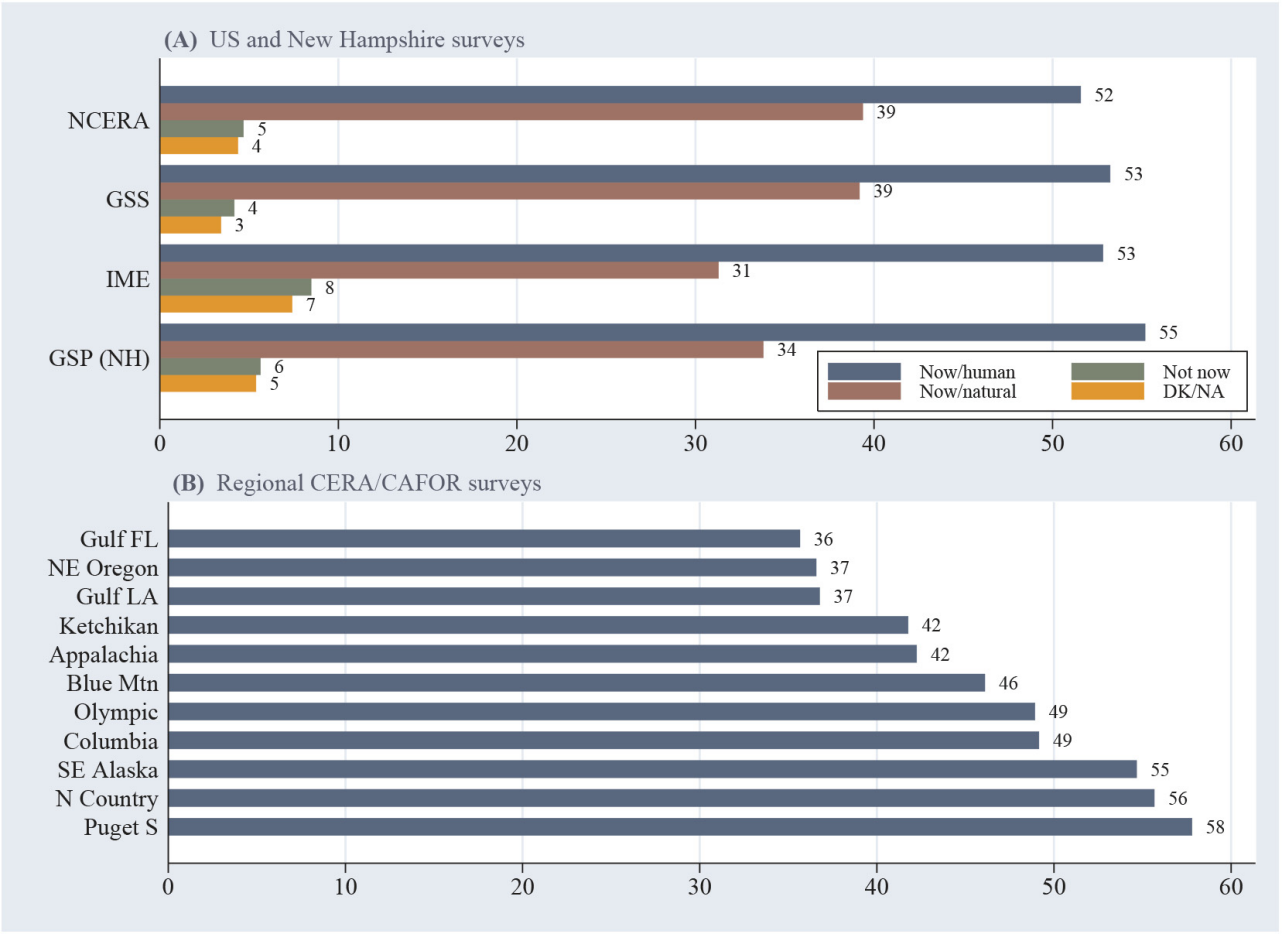
(A) Response percentages for climate-change question on 3 national and 21 statewide New Hampshire surveys; (B) percentage choosing the now/human response on 11 CERA/CAFOR surveys. Respondents who said they do not know, or gave no answer, are categorized as DK/NA in (A).

**Fig 2:**
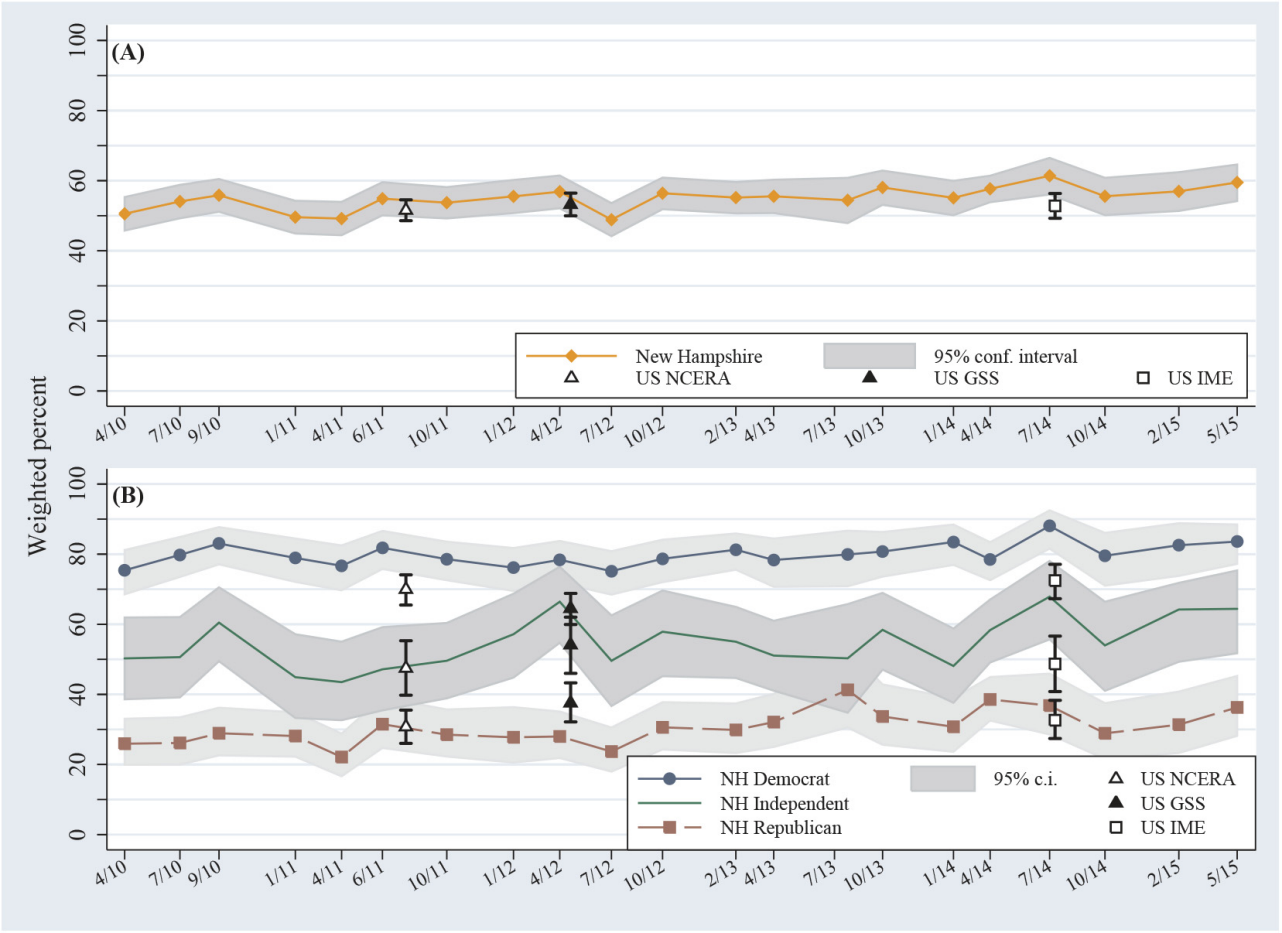
(A) Now/human response by date of survey, and (B) broken down by political party, spring 2010 to spring 2015. Surveys graphed at median interview dates, and shown with 95% confidence intervals.

The Stata 14.0 statistical program is employed for data management, analysis and graphing [21]. **Figs 1**and **2**chart response percentages calculated using probability weights as described above. Ninety-five percent confidence intervals appear with each data point in the time plots of Fig 2.

To quantify and test multiple predictors of now/human responses to the climate question, **Table 2**estimates five weighted logistic regression models. Such models are commonly employed with the categorical dependent variables of survey data. If *P*(*y*_*i*_ = 1) is the conditional probability of a now/human response by the *i*th individual, the odds of such a response are defined as *O*(*y*_*i*_ = 1) = *P*(*y*_*i*_ = 1)/*P*(*y*_*i*_ ≠1). Logistic regression models the conditional log odds as a linear function of *m* predictor variables *x*_1*i*_, *x*_2*i*_, …, *x*_m*i*_:

$$\text{ln}[O(y_{i}\;=\;1)]\;=\;\text{ln}\;\left[\frac{P(y_{i}\;=\;1)}{P(y_{i}\;=\;0)}\right]\;=\;\beta_{0}+\;\beta_{1}x_{1i}+\beta_{2}x_{2i}+\cdots +\;\beta_{m}x_{mi}$$

The β coefficients are estimated by maximum likelihood.

**Table 2:** Individual characteristics (all surveys), and county (CERA/CAFOR) or season, daily temperature anomaly and year (GSP), as predictors of belief that climate change is happening now, caused mainly by human activities. Odds ratios from weighted logistic regression.

	GSS	NCERA	IME	CERA	GSP
	national	national	national	regional	state
*Age*	0.995	0.987**	0.985***	0.983***	0.985***
*Gender*	1.116	1.129	1.074	1.213**	1.334***
*Education*	1.210**	1.249**	1.218**	1.205***	1.202***
*Party*	0.566***	0.482***	0.427***	0.425***	0.389***
*Education×party*	0.821**	0.785**	0.850	0.748***	0.780***
*County* (CERA)	. . .	. . .	. . .	(p < .001)	. . .
*Temperature* (GSP)	. . .	. . .	. . .	. . .	1.018*
*Season* (GSP)					
Winter	. . .	. . .	. . .	. . .	. . .
Spring	. . .	. . .	. . .	…	1.026
Summer	. . .	. . .	. . .	. . .	1.145
Fall	. . .	. . .	. . .	. . .	1.028
*Year* (GSP)	. . .	. . .	. . .	. . .	1.067***
estimation sample	1,242	1,714	960	11,554	10,567

* *p* < .05

** *p* < 0.1

*** *p* < .001

Exponentiating the estimated β coefficients, *e* ^*β*^, obtains odds ratios interpretable as multiplicative effects on *O*(*y*_*ij*_ = 1). Odds ratios greater than 1.0 represent “positive” effects, meaning that higher values of an *x* variable are associated with higher odds that *y* = 1. Odds ratios below 1.0 represent “negative” effects, meaning that higher *x* values are associated with lower odds that *y* = 1.

The *x* variables or predictors for all models in Table 2 include respondent *age* (in years), *gender* (0 male, 1 female), *education* (–1 high school or less, 0 some college or technical school, 1 college graduate, 2 postgraduate) and political *party* (–1 Democrat, 0 Independent, 1 Republican). Under this coding, when *education×party* interaction terms are present the main effects of *education* represent its effects when *party* = 0 (Independents). Similarly the main effects of *party* represent its effects when *education* = 0 (some college or technical school).

The CERA/CAFOR model in the fourth column of Table 2 pools data from 11 regional CERA or CAFOR surveys representing 38 different counties or occasions (see Table 1). Previous analysis found substantial county-to-county variation [22], so intercept dummy variables (0,1 indicators) for counties are included among the predictors. To represent 38 counties we need one intercept and 37 dummy variables, but for readability these 38 coefficients are not listed in the table. Instead, an adjusted Wald test for all of them together confirms significant (*p* < .001) place-to-place variation.

The GSP model in the fifth column of Table 2 pools data from 21 New Hampshire surveys, 2010–2015. Interview-day temperature anomaly, season and year are included among the predictors. A statewide temperature index (mean 0.9°C, range –11.1 to +14.6°C) is defined as the mean of anomalies (departures from 1981–2010 daily normals) across the state’s four continuing US Historical Climatology Network stations (Durham, Keene, Hanover and First Connecticut Lake). Season is represented by three dummy variables with winter as the base category. Including *year* among the predictors tests for a time trend.

**Fig 3:**
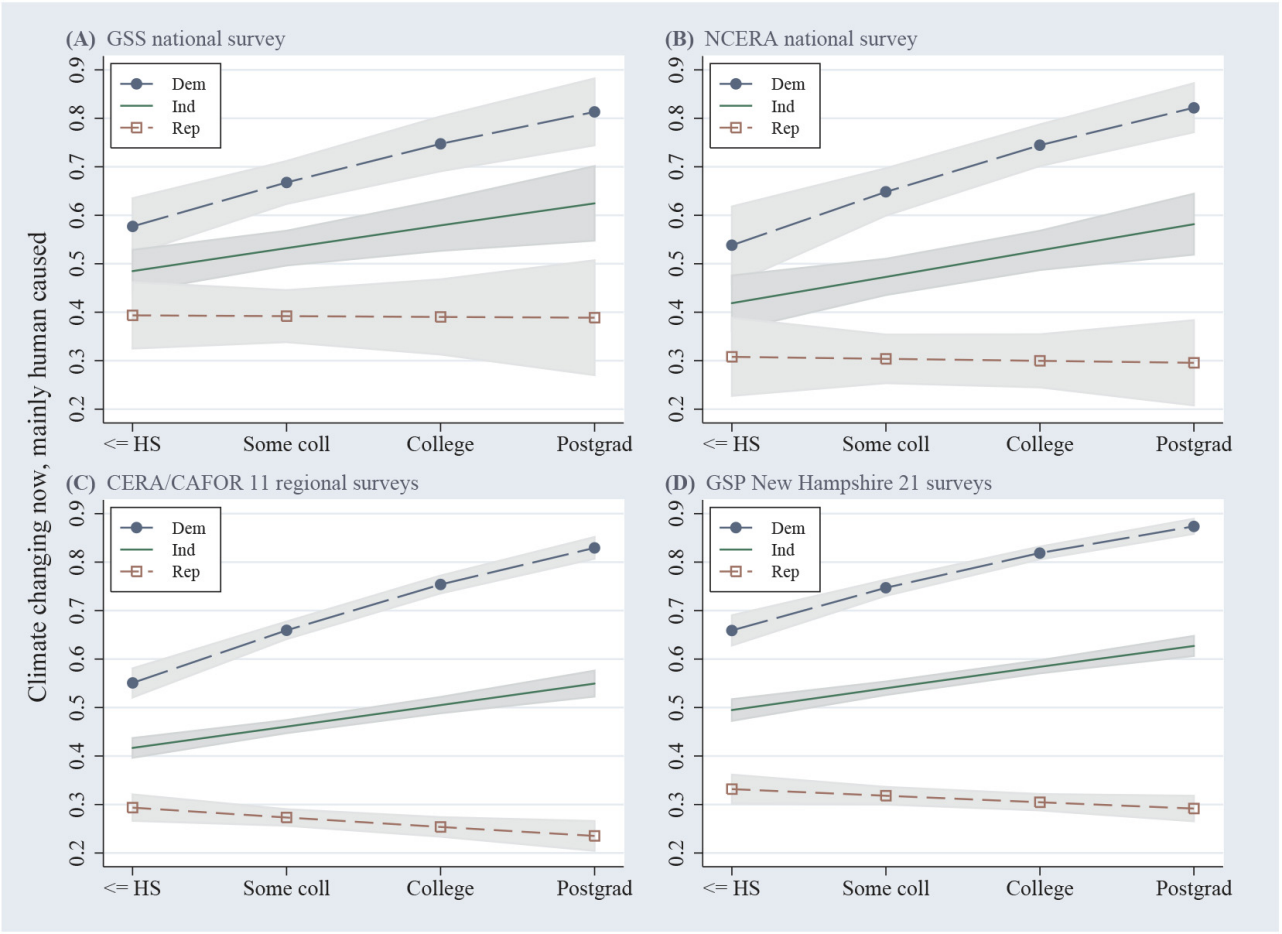
Probability of now/human response in GSS, NCERA, CERA/CAFOR and GSP surveys as a function of education, by political identification. Adjusted marginal plots with 95% confidence intervals calculated from the logistic regression models in Table 2.

Four significant *education*×*party* interaction effects from Table 2 are visualized as adjusted marginal plots [23] in **Fig 3**. Curves depict the predicted probability of a now/human response as a function of respondent education and political party identification, adjusted for all the other predictors in each model.

## Results

### Climate-change beliefs across 35 surveys

Three nationwide US surveys with diverse sampling and interview methods find 52% (in 2011) or 53% (in 2012 and 2014) agreement with the scientific consensus that human activities are now changing Earth’s climate (Fig 1A). A series of 21 statewide New Hampshire surveys over 2010 to 2015 runs a few points higher than the national surveys overall (55%). On these surveys a substantial minority (31 to 39%) concede that climate change is happening, but caused mainly by natural forces. Few (3 to 8%) say they believe climate change is not happening, or decline to express an opinion. In social, cognitive or political terms, the now/human and now/natural respondents prove distinct, whereas now/natural and not now respondents are less distinct [14]. Survey questions that simply ask whether global warming/climate change is happening, without specifying a cause, confuse two opposing viewpoints—in effect, grouping some of the now/natural responses together with now/human.

The CERA and CAFOR surveys target small and often rural clusters of counties, in regions selected for a variety of separate projects. Agreement with the scientific consensus ranges from 36 to 58% across these 11 surveys (Fig 1B). The regions studied include growing amenity-rich or near-urban areas, others dependent on coal or oil production, and still others with declining traditional resources such as forestry. Details of local environment and society help to understand place-to-place variations in climate and other environmental perceptions [18][19][24][25].

### Tracked over time

Fig 2A tracks the percentage of now/human responses on nationwide and New Hampshire surveys over time, and their 95% confidence intervals. The different surveys line up surprisingly well, with New Hampshire results a few points higher. Fig 2A gives a visual impression of slight upward drift, to be tested by the *year* coefficient in Table 2. In the New Hampshire time line we see no sign of a lasting impact from Hurricane Sandy, which brushed this state but caused no disaster there in late October 2012 (between our October 2012 and January 2013 surveys).

The placid surface of Fig 2A covers a deep partisan divide (Fig 2B). Overall around 80% of New Hampshire Democrats, 55% of Independents, and 31% of Republicans agree with the scientific consensus that climate change is happening now, caused mainly by human activities. This partisan gap is one of the largest in questions asked on our surveys. The gap is somewhat greater in New Hampshire than nationally, partly reflecting a higher proportion of college graduates who, as will be seen, tend to be most polarized on this issue. For GSS the partisan gap is just 27 points, but even that is wider than historically polarizing abortion or gun control questions asked on the same survey. Surveys using different questions suggest that partisan gaps in climate beliefs have widened over the past decade [9][26].

### Individual-level predictors

Political orientation and education dominate other characteristics in predicting individual responses. Moreover, politics moderates the effects of education. Table 2 quantifies these effects in logistic regression models that predict odds of a now/human response to the climate question. For the common individual-level predictors—*age*, *gender*, *education*, political *party* and *education*×*party*—these five analyses obtain remarkably consistent results.

*Age* effects are significant for every model except GSS, and all have odds ratios below 1, meaning that older respondents are less likely to agree with the scientific consensus. For example, an odds ratio of 0.985 (IME) indicates that the odds favoring a now/human response to the climate question are multiplied by 0.985, or decrease by 1.5%, with each one-year increase in age (if other predictors stay the same). With a 10-year increase in age, the odds are multiplied by 0.985^10^ = 0.860, or decrease about 14%.

In the CERA/CAFOR and GSP data women are significantly more likely to agree with the consensus, as shown by odds ratios above 1. The CERA/CAFOR odds ratio, 1.213, tells us that odds favoring a now/human response are about 21% higher for women than for men, other things being equal.

The main effects of *education*, significant across all of these models, suggest that among Independents (*party* = 0) the odds of belief in anthropogenic climate change increase by 20 to 25% (are multiplied by 1.202 to 1.249) with each step in *education*. Significant *education×party* interactions, however, indicate that the effects of education change with political party. Like the main effects of *education*, the magnitude of *education×party* interactions is roughly consistent (odds ratios from 0.748 to 0.850) across different datasets. Adjusted marginal plots in Fig 3 visualize the significant interactions in terms of probability. Among Democrats and Independents, probability of a now/human response rises with education. Among Republicans, however, this probability slightly declines with education. Better-educated Democrats and Republicans thus stand farther apart.

### Place-to-place variation

The CERA/CAFOR surveys covered 35 different counties, and re-surveyed three of them on two different occasions (2011 and 2014), for a total of 38 county/occasions. Earlier work found substantial place-to-place variation [22], motivating our inclusion of 37 intercept dummy variables in the regression model. As Table 2 notes these county/occasion indicators help to predict individual-level climate beliefs. An adjusted Wald test finds that the county indicators collectively have significant impacts.

Place-to-place variations can themselves be a focus of research. Studies using other dependent variables have found broad structural effects, as from unemployment or population growth rates, alongside other effects reflecting local circumstances such as the importance of coal mining in rural Kentucky or the experience of warming winters in northern New England [18][19][24][25].Our focus here has been on individual-characteristic effects that prove stable across many different surveys. This includes the CERA/CAFOR surveys where, after adjusting for the significant place-to-place variation, we find substantially the same individual effects (from *age*, *gender*, *education*, *party* and *education*×*party*) seen in other surveys.

### Temporal variation

The New Hampshire GSP interviews were conducted on 217 different days over 2010–2015. Temporal variation across this series of 21 surveys is much less than the spatial variation across the 11 regional CERA/CAFOR surveys, but it does display several patterns. Temperature anomalies on the interview day show a weak though significant effect on climate-change beliefs. Temperature effects prove intermittent within subsets of these data, however, marking them as not robust compared with individual and place effects.

The odds of belief in anthropogenic climate change are about 14% higher (multiplied by 1.145) in summer than winter. None of the seasonal effects are statistically significant, however. On the other hand, a slight upward trend in now/human responses, subjectively visible in Fig 2A, is more formally supported by a significant odds ratio for *year* in predicting the individual responses (Table 2). With each additional year, odds favoring a now/human response rise about 7% (multiplied by 1.67)—other things being equal.

## Discussion and Conclusions

### Politics and education effects

The most striking result here is the stability of public beliefs about anthropogenic climate change. That holds across different surveys (Fig 1A) and over a five-year time span (Fig 2A), although not across places (Fig 1B). General stability is anchored by wide, persistent political divisions (Fig 2B). Effects from individual age, gender, education and political party manifest as similar odds ratios on many different surveys (Table 2). Very similar *education*×*party* interaction effects occur in most of these surveys as well. In social research, interaction effects in multivariate models frequently prove to be sample-specific, so the degree of replication seen in Fig 3 is extraordinary.

Political identity dominates other background characteristics in predicting individual climate-change beliefs. Politics moderate effects from education, the second-strongest predictor. Agreement with the scientific consensus increases with education among Democrats and Independents (or liberals and moderates), but stays level or declines with education among Republicans (or conservatives). Similar interactions were first tested with different climate variables in 2006 GSS data [27] and subsequently replicated on other regional [24][28] and nationwide [9][29] surveys. Variations on this pattern include objectively-assessed science knowledge [30], numeracy [31] or self-assessed understanding [9][28] in place of education; and measures of ideology [9][27][28] or culture [31] in place of political party. Some other environment-related questions exhibit interactions of the same type [18][19][32].

Common explanations for the pattern invoke greater awareness among educated individuals about the views of politicians and media they follow—the *elite cues* hypothesis [9][33][34][35]. More educated or information-rich individuals also could be more effective in seeking out and retaining information that accords with their prejudices—as described by *biased assimilation* [9][36][37], *motivated skepticism* [38] and related hypotheses [26][39][40]. These explanations all hinge on the active, motivated acceptance/rejection of information, a major complication to the simpler *information deficit* hypothesis that people express low concern about scientifically-identified problems because they lack information that scientists could provide [41]. With regard to climate change many people assert that they are well informed, although their sense of understanding may come from politics rather than science [32].

### Place and temporal effects

Place-to-place variations can be substantial (Fig 1B). Other studies have found both systematic and idiosyncratic explanations for such place effects, reflecting characteristics of the local economy, history, environment and culture.[18][19][25].

Temporal variations over the years studied here have been smaller (Fig 2A), with only weak seasonal and daily temperature effects. The latter finding fits the mixed conclusions of previous research, in which some authors report effects from ambient conditions [42][43][44], weather [45][46][47][48][49][50] or climate trends [24][29]. Other studies, however, find minor or nonexistent effects from weather or climate [51][52]. These inconsistent results suggests that weather or climate effects tend to be minor and contingent, in contrast to the strong, ubiquitous effects of political orientation.

The New Hampshire time series was initiated to monitor possible changes in public agreement with the scientific consensus on climate change. The relative *lack* of change was an early, unexpected discovery. As the series lengthens, however, we see evidence of upward drift. Overall, New Hampshire public acceptance of anthropogenic climate change moved up about five points, from 53% in 2010 to 58% in 2015. This small but statistically significant (Table 2) drift roughly agrees with yearly nationwide results based on other survey questions [4]. That agreement on trends incidentally provides further encouragement for viewing the New Hampshire series as a proxy. Despite upward movement, both New Hampshire and national public opinion falls far short of the 97% consensus among climate scientists.

### Future research

The basic climate-change question offers currency, simplicity and unambiguous interpretation—whether individuals personally agree with the central point of scientific consensus on this globally important issue. As the examples here show, the question adapts readily to diverse survey instruments, opening possibilities for temporal, geographic and social-group comparisons. One planned future application is the 2016 General Social Survey, which offers an impressive range of sociological covariates. We also expect further regional surveys along the lines of CERA and CAFOR, investigating local variations. Finally, the same climate question has proven useful at smaller scales, in the benchmark and evaluation stages of education activities that are in progress but not described here.

The quarterly resolution and increasingly long run of the New Hampshire time series provides a unique platform to detect and characterize future change. To date it has shown only minor fluctuations around a slow upward drift. Seemingly large external events including an election and nearby hurricane had no detectable effects, but the possibility remains that cumulative or more extreme political, economic or climate-related events could have greater impact. With or without dramatic impacts, the series provides a monitoring system for the *shape* of any changes in public acceptance—whether abrupt or gradual, ephemeral or lasting.

## Supporting Information

S1 File National CERA Survey (NCERA 2011) variable list (pdf format). (PDF)

S2 File NCERA 2011 and CERA/CAFOR 2010–2011 surveys technical report (pdf format). (PDF)

S3 File Methodology Statement and Topline for iMediaEthics Poll on Climate Change 2014, conducted by Princeton Survey Research Associates International (pdf format). (PDF)

S4 File Stata graphing commands and statistical tables (pdf format). (PDF)

S1 Dataset NCERA 2011 national survey (Stata 12 format) (DTA)

S2 Dataset iMediaEthics 2014 national survey (Stata 12 format) (DTA)

S3 Dataset Climate change belief responses from many surveys (Stata 12 format). (DTA)

S4 Dataset Weighted percentages responding that climate change is happening now, mainly caused by human activities, on many surveys (Stata 12 format). (DTA)

S5 Dataset Weighted percentages responding that climate change is happening now, mainly caused by human activities, by political party identification on many surveys (Stata 12 format). (DTA)

S6 Dataset Climate change now/human responses from many surveys, by political party (Stata 12 format). (DTA)
