# Supplementary material for: Tracking Public Beliefs About Anthropogenic Climate Change
Source: PLoS One. 2015 Sep 30;10(9):e0138208. doi: 10.1371/journal.pone.0138208 (PMC4589389; doi:10.1371/journal.pone.0138208)
Supplement: S1 File — (PDF) [file pone.0138208.s001.pdf]

S1 File

Lawrence Hamilton
Carsey School of Public Policy
University of New Hampshire

July 2, 2015

National CERA Survey (NCERA)

50-state telephone survey conducted July–August 2011

Citations:

Hamilton, L.C. 2012. "Did the Arctic ice recover? Demographics of true and false climate facts." *Weather, Climate, and Society* 4(4):236–249. doi: 10.1175/WCAS-D-12-00008.1

Hamilton, L.C., J. Hartter, F. Stevens, R.G. Congalton, M. Ducey, M. Campbell, D. Maynard and M. Staunton. 2012. "Forest views: Shifting attitudes toward the environment in northeast Oregon." Durham, NH: Carsey Institute. http://scholars.unh.edu/carsey/162/

For more information:

Keirns, T. 2011. "Community and Environment in Rural America (CERA) Survey."

```
. describe

Contains data from C:\data\NCERA_2011_04.dta
  obs:         2,006                          National CERA survey, August 2011
 vars:            60                          1 May 2015 18:37
 size:       138,414
--------------------------------------------------------------------------------
              storage   display    value
variable name   type    format     label      variable label
--------------------------------------------------------------------------------
id_ncera        int     %9.0g                 National CERA case ID number
month           byte    %9.0g                 Month of interview
day             byte    %9.0g                 Day of interview
year            int     %9.0g                 Year of interview
state2          str2    %9s                   State abbreviation -- self
                                                reported
region4         byte    %9.0g      reg4       Census Region (4)
region9         byte    %12.0g     reg9       Census Division (9)
nonmetro        byte    %9.0g      nonmet     Metropolitan (0) or
                                                nonmetropolitan (1) county
ncerawt         float   %9.0g                 N-CERA survey wt --
                                                adults/age/race2/sex/reg9/nonmet
                                                > ro
sex2            byte    %9.0g      sex2       Gender
age             byte    %8.0g      age        Age in years
agegroup        byte    %9.0g      agegroup   Age group
sprawl2         byte    %8.0g      yesno      Problem: too-rapid development,
                                                growth, sprawl
popdec          byte    %8.0g      yesno      Problem: pop declining as people
                                                move away
jobopp          byte    %8.0g      yesno      Problem: lack of job opportunities
poverty         byte    %8.0g      yesno      Problem: poverty or homelessness
drugm           byte    %8.0g      yesno      Problem: manufacturing or sales
                                                illegal drugs
afford          byte    %8.0g      yesno      Problem: lack of affordable
```

				housing
recopp	byte	%8.0g	yesno	Problem: lack of recreational opportunities
leav	byte	%8.0g	yesno	Expect to move away in next 5 years
teen	byte	%8.0g	teen	Advise teen to stay or move away
foodcost2	byte	%9.0g	yesno	Willing to pay more for local food
envund2	byte	%10.0g	unders	Understanding: environmental, nat resource issues
envund2d	byte	%9.0g	yesno	Understand environment moderate/great deal
rules2	byte	%9.0g	rules2	Have conservation rules here been good?
wind2	byte	%9.0g	wind2	Oil drilling or renewable energy for future
oceanpol2	byte	%14.0g	oceanp2	Env problem: pollution of ocean
overfish2	byte	%14.0g	overf2	Env problem: overfishing of ocean
trusttv2	byte	%9.0g	trust	Env info trust: TV network news
trustsci2	byte	%9.0g	trust	Env info trust: scientists
optimist	byte	%12.0g	optimist	In 10 years community will be ...
race	byte	%18.0g	race	Respondent race
race2	byte	%8.0g	race2	Race (nonwhite)
employ	byte	%14.0g	employ	Employment status past year
party8	byte	%22.0g	party8	Political party (8)
party3	byte	%9.0g	party3	Political party (3)
relatt	byte	%14.0g	relatt	Religious service attendence
educ	byte	%13.0g	educ	Level of education (7)
educ4	byte	%17.0g	educ4	Level of education (4)
college	byte	%9.0g	yesno	College graduate
income6a	float	%9.0g	inc6a	Total household income (6+)
cell	byte	%9.0g	yesno	Survey done on cell phone, only phone they have
warmund2	byte	%10.0g	unders	Understanding: warming, climate change issues
warmop2	byte	%11.0g	warmop2	Personal belief about climate change
warmsci2	byte	%12.0g	warmsci2	Think scientists agree about climate change
warmund2d	byte	%9.0g	yesno	Understand climate moderate/great deal
warmop2d	byte	%9.0g	yesno	Personally believe climate now/human
warmsci2d	byte	%9.0g	yesno	Scientists agree climate now/human
warmice2	byte	%9.0g	warmice2	Area of late-summer Arctic ice v 30 yrs ago
warmice2d	byte	%9.0g		Area of Arctic sea ice less than 30 years ago
warmco22	byte	%10.0g	warmco22	Concentration of CO2 in atmosphere
warmgre2	byte	%13.0g	warmgre2	Meaning of 'greenhouse effect'
rightice	byte	%9.0g	yesno	Arctic ice question correct
rightco2	byte	%9.0g	yesno	CO2 concentration question correct
rightgre	byte	%9.0g	yesno	Greenhouse effect meaning correct
future2	byte	%13.0g	future3	In future, use resources for jobs or conservation?
landown2	byte	%13.0g	landown2	Land use views
future2d	byte	%9.0g	yesno	In future, conserve resources
rules2d	byte	%9.0g	yesno	Environmental rules good here
trustsci2d	byte	%9.0g	yesno	Trust scientists for environmental info

----

Sorted by: id_ncera

. summarize

Variable	Obs	Mean	Std. Dev.	Min	Max
id_ncera	2,006	1003.5	579.2266	1	2006
month	2,005	7.587032	.4924899	7	8
day	2,005	15.00499	8.260116	1	31
year	2,005	2011	0	2011	2011
state2	0				
region4	2,006	2.609172	1.000646	1	4
region9	2,006	5.036889	2.437986	1	9
nonmetro	2,006	.3639083	.4812427	0	1
ncerawt	2,006	1	.9295616	.2445446	3.912713
sex2	2,006	.5922233	.4915438	0	1
age	1,982	54.84157	17.39737	18	95
agegroup	1,982	3.65338	1.293183	1	5
sprawl2	1,948	.2356263	.4244986	0	1
popdec	1,967	.2633452	.4405601	0	1
jobopp	1,935	.7875969	.4091143	0	1
poverty	1,930	.5756477	.4943724	0	1
drugm	1,833	.6350245	.4815547	0	1
afford	1,896	.4746835	.4994904	0	1
recopp	1,964	.3258656	.4688167	0	1
leav	1,940	.1721649	.3776211	0	1
teen	1,726	.5341831	.4989747	0	1
foodcost2	1,892	.7034884	.45684	0	1
envund2	2,006	3.068794	.7272764	1	4
envund2d	2,006	.8185444	.3854913	0	1
rules2	2,006	2.232802	.6862677	1	3
wind2	2,006	1.845962	.5157868	1	3
oceanpol2	2,006	2.5	.7283021	1	3
overfish2	2,006	2.086241	.8615014	1	3
trusttv2	2,006	1.749751	.7133829	1	3
trustsci2	2,006	2.297109	.7430218	1	3
optimist	1,939	1.078907	.6848377	0	2
race	1,963	1.354559	.9442208	1	6
race2	1,963	.1711666	.3767504	0	1
employ	1,980	2.194444	1.097136	1	4
party8	1,861	4.06878	2.294828	1	8
party3	1,747	1.941042	.9065522	1	3
relatt	1,959	1.885656	1.383617	0	4
educ	1,977	4.980779	1.587411	1	7
educ4	1,977	2.367223	1.090861	1	4
college	2,006	.448654	.4974806	0	1
income6a	1,641	3.300122	1.557034	1	6
cell	2,006	.0358923	.1860681	0	1
warmund2	2,006	2.884845	.8133546	1	4
warmop2	2,006	2.284148	.8439416	0	3
warmsci2	2,006	1.392822	.6716402	0	2
warmund2d	2,006	.7168495	.4506413	0	1
warmop2d	2,006	.4725823	.4993722	0	1
warmsci2d	2,006	.499003	.5001237	0	1
warmice2	2,006	1.702891	1.106076	1	4
warmice2d	2,006	.6605184	.4736514	0	1

warmco22	2,006	2.817547	.7746682	1	4
warmgre2	2,006	2.188933	.9940959	1	4
rightice	2,006	.6605184	.4736514	0	1
rightco2	2,006	.5942173	.4911653	0	1
rightgre	2,006	.5104686	.500015	0	1
future2	1,865	2.020375	.8980265	1	3
landown2	1,750	.3862857	.4870366	0	1
future2d	2,006	.384347	.4865618	0	1
rules2d	2,006	.3788634	.485225	0	1
trustsci2d	2,006	.4685942	.4991371	0	1

```
. codebook

--------------------------------------------------------------------------------
id_ncera                                              National CERA case ID number
--------------------------------------------------------------------------------

                  type:  numeric (int)

                 range:  [1,2006]                     units:  1
         unique values:  2,006                    missing .:  0/2,006

                  mean:    1003.5
              std. dev:   579.227

           percentiles:        10%       25%       50%       75%       90%
                               201       502    1003.5      1505      1806

--------------------------------------------------------------------------------
month                                                          Month of interview
--------------------------------------------------------------------------------

                  type:  numeric (byte)

                 range:  [7,8]                        units:  1
         unique values:  2                        missing .:  1/2,006

            tabulation:  Freq.  Value
                           828  7
                         1,177  8
                             1  .

--------------------------------------------------------------------------------
day                                                              Day of interview
--------------------------------------------------------------------------------

                  type:  numeric (byte)

                 range:  [1,31]                       units:  1
         unique values:  31                       missing .:  1/2,006

                  mean:    15.005
              std. dev:   8.26012

           percentiles:        10%       25%       50%       75%       90%
                                 3         9        15        20        28

--------------------------------------------------------------------------------
year                                                            Year of interview
--------------------------------------------------------------------------------

                  type:  numeric (int)

                 range:  [2011,2011]                  units:  1
         unique values:  1                        missing .:  1/2,006

            tabulation:  Freq.  Value
                         2,005  2011
                             1  .

--------------------------------------------------------------------------------
state2                                         State abbreviation -- self reported
--------------------------------------------------------------------------------
```

```
                type:  string (str2)

       unique values:  51                       missing "":  0/2,006

            examples:  "FL"
                       "MD"
                       "NM"
                       "TN"

-------------------------------------------------------------------------------
region4                                                      Census Region  (4)
-------------------------------------------------------------------------------

                type:  numeric (byte)
               label:  reg4

               range:  [1,4]                        units:  1
       unique values:  4                        missing .:  0/2,006

          tabulation:  Freq.   Numeric  Label
                         344         1  Northeast
                         517         2  Midwest
                         724         3  South
                         421         4  West

-------------------------------------------------------------------------------
region9                                                    Census Division  (9)
-------------------------------------------------------------------------------

                type:  numeric (byte)
               label:  reg9

               range:  [1,9]                        units:  1
       unique values:  9                        missing .:  0/2,006

          tabulation:  Freq.   Numeric  Label
                         123         1  New England
                         221         2  Mid Atlantic
                         322         3  E N Central
                         195         4  W N Central
                         370         5  S Atlantic
                         149         6  E S Central
                         205         7  W S Central
                         175         8  Mountain
                         246         9  Pacific

-------------------------------------------------------------------------------
nonmetro                          Metropolitan (0) or nonmetropolitan (1) county
-------------------------------------------------------------------------------

                type:  numeric (byte)
               label:  nonmet

               range:  [0,1]                        units:  1
       unique values:  2                        missing .:  0/2,006

          tabulation:  Freq.   Numeric  Label
                       1,276         0  Metro
                         730         1  Nonmetro

-------------------------------------------------------------------------------
ncerawt                   N-CERA survey wt -- adults/age/race2/sex/reg9/nonmetro
-------------------------------------------------------------------------------
```

```
                  type:  numeric (float)

                 range:  [.24454455,3.9127128]        units:  1.000e-08
         unique values:  545                      missing .:  0/2,006

                  mean:          1
             std. dev:    .929562

           percentiles:        10%       25%       50%       75%       90%
                          .244545   .332281   .675804    1.2479   2.20046

--------------------------------------------------------------------------------
sex2                                                                      Gender
--------------------------------------------------------------------------------

                  type:  numeric (byte)
                 label:  sex2

                 range:  [0,1]                        units:  1
         unique values:  2                        missing .:  0/2,006

            tabulation:  Freq.   Numeric  Label
                           818         0  Male
                         1,188         1  Female

--------------------------------------------------------------------------------
age                                                                 Age in years
--------------------------------------------------------------------------------

                  type:  numeric (byte)
                 label:  age, but 77 nonmissing values are not labeled

                 range:  [18,95]                      units:  1
         unique values:  77                       missing .:  24/2,006

              examples:  39
                         52
                         61
                         71

--------------------------------------------------------------------------------
agegroup                                                               Age group
--------------------------------------------------------------------------------

                  type:  numeric (byte)
                 label:  agegroup

                 range:  [1,5]                        units:  1
         unique values:  5                        missing .:  0/2,006
       unique mv codes:  1                       missing .*:  24/2,006

            tabulation:  Freq.   Numeric  Label
                           198         1  18-29
                           211         2  30-39
                           298         3  40-49
                           648         4  50-64
                           627         5  65+
                            24        .a  DK/NA

--------------------------------------------------------------------------------
sprawl2                           Problem: too-rapid development, growth, sprawl
--------------------------------------------------------------------------------

                  type:  numeric (byte)
```

```
                label:  yesno

                range:  [0,1]                        units:  1
        unique values:  2                        missing .:  0/2,006
      unique mv codes:  1                       missing .*:  58/2,006

          tabulation:  Freq.   Numeric  Label
                       1,489         0  No
                         459         1  Yes
                          58        .a  DK/NA

-------------------------------------------------------------------------------
popdec                                  Problem: pop declining as people move away
-------------------------------------------------------------------------------

                 type:  numeric (byte)
                label:  yesno

                range:  [0,1]                        units:  1
        unique values:  2                        missing .:  0/2,006
      unique mv codes:  1                       missing .*:  39/2,006

          tabulation:  Freq.   Numeric  Label
                       1,449         0  No
                         518         1  Yes
                          39        .a  DK/NA

-------------------------------------------------------------------------------
jobopp                                          Problem: lack of job opportunities
-------------------------------------------------------------------------------

                 type:  numeric (byte)
                label:  yesno

                range:  [0,1]                        units:  1
        unique values:  2                        missing .:  0/2,006
      unique mv codes:  1                       missing .*:  71/2,006

          tabulation:  Freq.   Numeric  Label
                         411         0  No
                       1,524         1  Yes
                          71        .a  DK/NA

-------------------------------------------------------------------------------
poverty                                        Problem: poverty or homelessness
-------------------------------------------------------------------------------

                 type:  numeric (byte)
                label:  yesno

                range:  [0,1]                        units:  1
        unique values:  2                        missing .:  0/2,006
      unique mv codes:  1                       missing .*:  76/2,006

          tabulation:  Freq.   Numeric  Label
                         819         0  No
                       1,111         1  Yes
                          76        .a  DK/NA

-------------------------------------------------------------------------------
drugm                              Problem: manufacturing or sales illegal drugs
-------------------------------------------------------------------------------

                 type:  numeric (byte)
```

```
                label:  yesno

                range:  [0,1]                        units:  1
        unique values:  2                        missing .:  0/2,006
      unique mv codes:  1                       missing .*:  173/2,006

          tabulation:  Freq.   Numeric  Label
                         669         0  No
                       1,164         1  Yes
                         173        .a  DK/NA

--------------------------------------------------------------------------------
afford                                    Problem: lack of affordable housing
--------------------------------------------------------------------------------

                 type:  numeric (byte)
                label:  yesno

                range:  [0,1]                        units:  1
        unique values:  2                        missing .:  0/2,006
      unique mv codes:  1                       missing .*:  110/2,006

          tabulation:  Freq.   Numeric  Label
                         996         0  No
                         900         1  Yes
                         110        .a  DK/NA

--------------------------------------------------------------------------------
recopp                              Problem: lack of recreational opportunities
--------------------------------------------------------------------------------

                 type:  numeric (byte)
                label:  yesno

                range:  [0,1]                        units:  1
        unique values:  2                        missing .:  0/2,006
      unique mv codes:  1                       missing .*:  42/2,006

          tabulation:  Freq.   Numeric  Label
                       1,324         0  No
                         640         1  Yes
                          42        .a  DK/NA

--------------------------------------------------------------------------------
leav                                      Expect to move away in next 5 years
--------------------------------------------------------------------------------

                 type:  numeric (byte)
                label:  yesno

                range:  [0,1]                        units:  1
        unique values:  2                        missing .:  0/2,006
      unique mv codes:  1                       missing .*:  66/2,006

          tabulation:  Freq.   Numeric  Label
                       1,606         0  No
                         334         1  Yes
                          66        .a  DK/NA

--------------------------------------------------------------------------------
teen                                         Advise teen to stay or move away
--------------------------------------------------------------------------------

                 type:  numeric (byte)
```

```
                 label:  teen

                 range:  [0,1]                        units:  1
         unique values:  2                        missing .:  0/2,006
       unique mv codes:  1                       missing .*:  280/2,006

           tabulation:  Freq.   Numeric  Label
                          804         0  Stay
                          922         1  Move
                          280        .a  DK/NA

-------------------------------------------------------------------------------
foodcost2                                   Willing to pay more for local food
-------------------------------------------------------------------------------

                  type:  numeric (byte)
                 label:  yesno

                 range:  [0,1]                        units:  1
         unique values:  2                        missing .:  0/2,006
       unique mv codes:  1                       missing .*:  114/2,006

           tabulation:  Freq.   Numeric  Label
                          561         0  No
                        1,331         1  Yes
                          114        .a  DK/NA

-------------------------------------------------------------------------------
envund2                       Understanding: environmental, nat resource issues
-------------------------------------------------------------------------------

                  type:  numeric (byte)
                 label:  unders

                 range:  [1,4]                        units:  1
         unique values:  4                        missing .:  0/2,006

           tabulation:  Freq.   Numeric  Label
                           51         1  DK/nothing
                          313         2  Little
                        1,089         3  Moderate
                          553         4  Great deal

-------------------------------------------------------------------------------
envund2d                             Understand environment moderate/great deal
-------------------------------------------------------------------------------

                  type:  numeric (byte)
                 label:  yesno

                 range:  [0,1]                        units:  1
         unique values:  2                        missing .:  0/2,006

           tabulation:  Freq.   Numeric  Label
                          364         0  No
                        1,642         1  Yes

-------------------------------------------------------------------------------
rules2                                      Have conservation rules here been good?
-------------------------------------------------------------------------------

                  type:  numeric (byte)
                 label:  rules2
```

```
              range:  [1,3]                        units:  1
      unique values:  3                        missing .:  0/2,006

        tabulation:  Freq.   Numeric  Label
                       293         1  Bad here
                       953         2  No effect
                       760         3  Good here

--------------------------------------------------------------------------------
wind2                               Oil drilling or renewable energy for future
--------------------------------------------------------------------------------

                type:  numeric (byte)
               label:  wind2

               range:  [1,3]                        units:  1
       unique values:  3                        missing .:  0/2,006

          tabulation:  Freq.   Numeric  Label
                         445         1  Drilling
                       1,425         2  Renewable
                         136         3  DK/NA

--------------------------------------------------------------------------------
oceanpol2                                       Env problem: pollution of ocean
--------------------------------------------------------------------------------

                type:  numeric (byte)
               label:  oceanp2

               range:  [1,3]                        units:  1
       unique values:  3                        missing .:  0/2,006

          tabulation:  Freq.   Numeric  Label
                         281         1  Not problem/DK
                         441         2  Not serious
                       1,284         3  Serious

--------------------------------------------------------------------------------
overfish2                                     Env problem: overfishing of ocean
--------------------------------------------------------------------------------

                type:  numeric (byte)
               label:  overf2

               range:  [1,3]                        units:  1
       unique values:  3                        missing .:  0/2,006

          tabulation:  Freq.   Numeric  Label
                         665         1  Not problem/DK
                         503         2  Not serious
                         838         3  Serious

--------------------------------------------------------------------------------
trusttv2                                        Env info trust: TV network news
--------------------------------------------------------------------------------

                type:  numeric (byte)
               label:  trust

               range:  [1,3]                        units:  1
       unique values:  3                        missing .:  0/2,006

          tabulation:  Freq.   Numeric  Label
```

```
                  824         1  Don't
                  860         2  Unsure/DK
                  322         3  Trust

-------------------------------------------------------------------------------
trustsci2                                         Env info trust: scientists
-------------------------------------------------------------------------------

                  type:  numeric (byte)
                 label:  trust

                 range:  [1,3]                        units:  1
         unique values:  3                        missing .:  0/2,006

            tabulation:  Freq.   Numeric  Label
                           344         1  Don't
                           722         2  Unsure/DK
                           940         3  Trust

-------------------------------------------------------------------------------
optimist                                   In 10 years community will be ...
-------------------------------------------------------------------------------

                  type:  numeric (byte)
                 label:  optimist

                 range:  [0,2]                        units:  1
         unique values:  3                        missing .:  0/2,006
       unique mv codes:  1                       missing .*:  67/2,006

            tabulation:  Freq.   Numeric  Label
                           384         0  Worse place
                         1,018         1  About same
                           537         2  Better place
                            67        .a  DK/NA

-------------------------------------------------------------------------------
race                                                         Respondent race
-------------------------------------------------------------------------------

                  type:  numeric (byte)
                 label:  race

                 range:  [1,6]                        units:  1
         unique values:  6                        missing .:  0/2,006
       unique mv codes:  1                       missing .*:  43/2,006

            tabulation:  Freq.   Numeric  Label
                         1,627         1  White non-Hispanic
                           158         2  African American
                            80         3  Hispanic
                            38         4  Asian American
                            36         5  Native Am/AK/HI
                            24         6  other
                            43        .a  DK/NA

-------------------------------------------------------------------------------
race2                                                       Race (nonwhite)
-------------------------------------------------------------------------------

                  type:  numeric (byte)
                 label:  race2

                 range:  [0,1]                        units:  1
```

```
    unique values:  2                        missing .:  0/2,006
  unique mv codes:  1                       missing .*:  43/2,006

      tabulation:  Freq.   Numeric  Label
                   1,627         0  White
                     336         1  Nonwhite
                      43        .a  DK/NA

--------------------------------------------------------------------------------
employ                                              Employment status past year
--------------------------------------------------------------------------------

                type:  numeric (byte)
               label:  employ

               range:  [1,4]                        units:  1
       unique values:  4                        missing .:  0/2,006
     unique mv codes:  1                       missing .*:  26/2,006

          tabulation:  Freq.   Numeric  Label
                         787         1  Full time
                         270         2  Part time/year
                         674         3  Retired
                         249         4  Not employed
                          26        .a  DK/NA

--------------------------------------------------------------------------------
party8                                                      Political party (8)
--------------------------------------------------------------------------------

                type:  numeric (byte)
               label:  party8

               range:  [1,8]                        units:  1
       unique values:  8                        missing .:  0/2,006
     unique mv codes:  1                       missing .*:  145/2,006

          tabulation:  Freq.   Numeric  Label
                         375         1  Strong Democrat
                         232         2  Not very strong Dem
                         165         3  Ind, closer to Dem
                         306         4  Ind, closer to neither
                         198         5  Ind, closer to Rep
                         185         6  Not very strong Rep
                         286         7  Strong Republican
                         114         8  other
                         145        .a  DK/NA

--------------------------------------------------------------------------------
party3                                                      Political party (3)
--------------------------------------------------------------------------------

                type:  numeric (byte)
               label:  party3

               range:  [1,3]                        units:  1
       unique values:  3                        missing .:  0/2,006
     unique mv codes:  1                       missing .*:  259/2,006

          tabulation:  Freq.   Numeric  Label
                         772         1  Dem
                         306         2  Ind
                         669         3  Rep
                         259        .a  DK/NA
```

```
--------------------------------------------------------------------------------
relatt                                              Religious service attendence
--------------------------------------------------------------------------------

                  type:  numeric (byte)
                 label:  relatt

                 range:  [0,4]                        units:  1
         unique values:  5                        missing .:  0/2,006
       unique mv codes:  1                       missing .*:  47/2,006

            tabulation:  Freq.   Numeric  Label
                           432         0  Never
                           443         1  Few times/year
                           261         2  1-2/month
                           563         3  1/week
                           260         4  > 1/week
                            47        .a  DK/NA

--------------------------------------------------------------------------------
educ                                                    Level of education (7)
--------------------------------------------------------------------------------

                  type:  numeric (byte)
                 label:  educ

                 range:  [1,7]                        units:  1
         unique values:  7                        missing .:  0/2,006
       unique mv codes:  1                       missing .*:  29/2,006

            tabulation:  Freq.   Numeric  Label
                            17         1  <= 8th
                            77         2  Some HS
                           466         3  HS grad
                            93         4  Tech school
                           424         5  Some coll
                           514         6  College grad
                           386         7  Postgrad work
                            29        .a  DK/NA

--------------------------------------------------------------------------------
educ4                                                   Level of education (4)
--------------------------------------------------------------------------------

                  type:  numeric (byte)
                 label:  educ4

                 range:  [1,4]                        units:  1
         unique values:  4                        missing .:  0/2,006
       unique mv codes:  1                       missing .*:  29/2,006

            tabulation:  Freq.   Numeric  Label
                           560         1  HS or less
                           517         2  Tech/some college
                           514         3  College
                           386         4  Postgrad
                            29        .a  DK/NA

--------------------------------------------------------------------------------
college                                                       College graduate
--------------------------------------------------------------------------------

                  type:  numeric (byte)
                 label:  yesno
```

```
                  range:  [0,1]                        units:  1
          unique values:  2                        missing .:  0/2,006

            tabulation:  Freq.   Numeric  Label
                         1,106         0  No
                           900         1  Yes

-------------------------------------------------------------------------------
income6a                                             Total household income (6+)
-------------------------------------------------------------------------------

                   type:  numeric (float)
                  label:  inc6a, but 3 nonmissing values are not labeled

                  range:  [1,6]                        units:  .1
          unique values:  9                        missing .:  0/2,006
        unique mv codes:  1                       missing .*:  365/2,006

            tabulation:  Freq.   Numeric  Label
                           237         1  < $20k
                            16       1.5
                           360         2  $20-$40k
                           240         3  $40-60k
                            28       3.5
                           328         4  $60-$90k
                           276         5  $90-$160k
                            23       5.5
                           133         6  > $160k
                           365        .a  DK/NA

-------------------------------------------------------------------------------
cell                                Survey done on cell phone, only phone they have
-------------------------------------------------------------------------------

                   type:  numeric (byte)
                  label:  yesno

                  range:  [0,1]                        units:  1
          unique values:  2                        missing .:  0/2,006

            tabulation:  Freq.   Numeric  Label
                         1,934         0  No
                            72         1  Yes

-------------------------------------------------------------------------------
warmund2                              Understanding: warming, climate change issues
-------------------------------------------------------------------------------

                   type:  numeric (byte)
                  label:  unders

                  range:  [1,4]                        units:  1
          unique values:  4                        missing .:  0/2,006

            tabulation:  Freq.   Numeric  Label
                           112         1  DK/nothing
                           456         2  Little
                           989         3  Moderate
                           449         4  Great deal

-------------------------------------------------------------------------------
warmop2                                        Personal belief about climate change
-------------------------------------------------------------------------------
```

```
              type:  numeric (byte)
             label:  warmop2

             range:  [0,3]                        units:  1
     unique values:  4                        missing .:  0/2,006

        tabulation:  Freq.   Numeric  Label
                       132         0  DK/NA
                       114         1  Not now
                       812         2  Now/natural
                       948         3  Now/human

--------------------------------------------------------------------------------
warmsci2                            Think scientists agree about climate change
--------------------------------------------------------------------------------

              type:  numeric (byte)
             label:  warmsci2

             range:  [0,2]                        units:  1
     unique values:  3                        missing .:  0/2,006

        tabulation:  Freq.   Numeric  Label
                       213         0  DK/NA
                       792         1  Little agree
                     1,001         2  Most agree

--------------------------------------------------------------------------------
warmund2d                                Understand climate moderate/great deal
--------------------------------------------------------------------------------

              type:  numeric (byte)
             label:  yesno

             range:  [0,1]                        units:  1
     unique values:  2                        missing .:  0/2,006

        tabulation:  Freq.   Numeric  Label
                       568         0  No
                     1,438         1  Yes

--------------------------------------------------------------------------------
warmop2d                                   Personally believe climate now/human
--------------------------------------------------------------------------------

              type:  numeric (byte)
             label:  yesno

             range:  [0,1]                        units:  1
     unique values:  2                        missing .:  0/2,006

        tabulation:  Freq.   Numeric  Label
                     1,058         0  No
                       948         1  Yes

--------------------------------------------------------------------------------
warmsci2d                                    Scientists agree climate now/human
--------------------------------------------------------------------------------

              type:  numeric (byte)
             label:  yesno

             range:  [0,1]                        units:  1
     unique values:  2                        missing .:  0/2,006
```

```
           tabulation:  Freq.   Numeric  Label
                        1,005         0  No
                        1,001         1  Yes

--------------------------------------------------------------------------------
warmice2                            Area of late-summer Arctic ice v 30 yrs ago
--------------------------------------------------------------------------------

                 type:  numeric (byte)
                label:  warmice2

                range:  [1,4]                        units:  1
        unique values:  4                        missing .:  0/2,006

           tabulation:  Freq.   Numeric  Label
                        1,325         1  Less
                          240         2  Recovered
                          153         3  More
                          288         4  DK/NA

--------------------------------------------------------------------------------
warmice2d                         Area of Arctic sea ice less than 30 years ago
--------------------------------------------------------------------------------

                 type:  numeric (byte)

                range:  [0,1]                        units:  1
        unique values:  2                        missing .:  0/2,006

           tabulation:  Freq.  Value
                          681  0
                        1,325  1

--------------------------------------------------------------------------------
warmco22                                     Concentration of CO2 in atmosphere
--------------------------------------------------------------------------------

                 type:  numeric (byte)
                label:  warmco22

                range:  [1,4]                        units:  1
        unique values:  4                        missing .:  0/2,006

           tabulation:  Freq.   Numeric  Label
                          152         1  Decreasing
                          362         2  Same
                        1,192         3  Increasing
                          300         4  DK/NA

--------------------------------------------------------------------------------
warmgre2                                         Meaning of 'greenhouse effect'
--------------------------------------------------------------------------------

                 type:  numeric (byte)
                label:  warmgre2

                range:  [1,4]                        units:  1
        unique values:  4                        missing .:  0/2,006

           tabulation:  Freq.   Numeric  Label
                          480         1  Ozone hole
                        1,024         2  Heat-trapping
                          145         3  Pavement
                          357         4  DK/NA
```

```
-------------------------------------------------------------------------------
rightice                                          Arctic ice question correct
-------------------------------------------------------------------------------

                  type:  numeric (byte)
                 label:  yesno

                 range:  [0,1]                        units:  1
         unique values:  2                        missing .:  0/2,006

            tabulation:  Freq.   Numeric  Label
                           681         0  No
                         1,325         1  Yes

-------------------------------------------------------------------------------
rightco2                                   CO2 concentration question correct
-------------------------------------------------------------------------------

                  type:  numeric (byte)
                 label:  yesno

                 range:  [0,1]                        units:  1
         unique values:  2                        missing .:  0/2,006

            tabulation:  Freq.   Numeric  Label
                           814         0  No
                         1,192         1  Yes

-------------------------------------------------------------------------------
rightgre                                    Greenhouse effect meaning correct
-------------------------------------------------------------------------------

                  type:  numeric (byte)
                 label:  yesno

                 range:  [0,1]                        units:  1
         unique values:  2                        missing .:  0/2,006

            tabulation:  Freq.   Numeric  Label
                           982         0  No
                         1,024         1  Yes

-------------------------------------------------------------------------------
future2                      In future, use resources for jobs or conservation?
-------------------------------------------------------------------------------

                  type:  numeric (byte)
                 label:  future3

                 range:  [1,3]                        units:  1
         unique values:  3                        missing .:  0/2,006
       unique mv codes:  1                       missing .*:  141/2,006

            tabulation:  Freq.   Numeric  Label
                           733         1  Use resources
                           361         2  Both equal
                           771         3  Conserve
                           141        .a  DK/NA

-------------------------------------------------------------------------------
landown2                                                         Land use views
-------------------------------------------------------------------------------

                  type:  numeric (byte)
```

```
                label:  landown2

                range:  [0,1]                        units:  1
        unique values:  2                        missing .:  0/2,006
      unique mv codes:  1                       missing .*:  256/2,006

          tabulation:  Freq.   Numeric  Label
                       1,074         0  Govt regulate
                         676         1  Owners free
                         256        .a  DK/NA

-------------------------------------------------------------------------------
future2d                                          In future, conserve resources
-------------------------------------------------------------------------------

                 type:  numeric (byte)
                label:  yesno

                range:  [0,1]                        units:  1
        unique values:  2                        missing .:  0/2,006

          tabulation:  Freq.   Numeric  Label
                       1,235         0  No
                         771         1  Yes

-------------------------------------------------------------------------------
rules2d                                           Environmental rules good here
-------------------------------------------------------------------------------

                 type:  numeric (byte)
                label:  yesno

                range:  [0,1]                        units:  1
        unique values:  2                        missing .:  0/2,006

          tabulation:  Freq.   Numeric  Label
                       1,246         0  No
                         760         1  Yes

-------------------------------------------------------------------------------
trustsci2d                                Trust scientists for environmental info
-------------------------------------------------------------------------------

                 type:  numeric (byte)
                label:  yesno

                range:  [0,1]                        units:  1
        unique values:  2                        missing .:  0/2,006

          tabulation:  Freq.   Numeric  Label
                       1,066         0  No
                         940         1  Yes
```
