# Supplementary material for: Tracking Public Beliefs About Anthropogenic Climate Change
Source: PLoS One. 2015 Sep 30;10(9):e0138208. doi: 10.1371/journal.pone.0138208 (PMC4589389; doi:10.1371/journal.pone.0138208)
Supplement: S2 File — (PDF) [file pone.0138208.s002.pdf]

S2 File

COMMUNITY AND ENVIRONMENT IN RURAL AMERICA (CERA) SURVEY

Conducted for:

The Carsey Institute,
University of New Hampshire

Prepared by:

Tracy Keirns

The Survey Center
University of New Hampshire

August 2011

TECHNICAL REPORT

The University of New Hampshire Survey Center conducted telephone surveys of adults in several regions of the United States as part of the UNH Carsey Institute Community and Environment in Rural America (CERA) Survey in 2008 through 2010. The project was funded with grants from the Ford Foundation, the Kellogg Foundation, and the Tillotson Foundation.

The following regions were included in the project:

- Choctaw, Marengo, and Wilcox Counties, Alabama (2007)
- Chaffee County, Colorado (2007)
- Cheyenne, Jewell, Republic and Smith Counties, Kansas (2007)
- Letcher and Harlan Counties, Kentucky (2007)
- Coahoma and Tunica Counties, Mississippi (2007)
- Clatsop County, Oregon and Pacific County, Washington (2007)
- Alger, Chippewa, Luce, Mackinac, and Schoolcraft Counties in Upper Peninsula, Michigan (2008)
- Hancock and Washington Counties in Maine (2009)
- Oxford County, Maine (2007, 2010)
- Coos County, New Hampshire (2007, 2010)
- Essex County, Vermont (2010)
- Plaquemines and Terrebonne Parishes, Louisiana (2010)
- Bay, Franklin and Guld Counties, Florida (2010)
- Alaska Towns (2010)
- Clallam, Gray's Harbor and Jefferson Counties, Washington (2010)
- Haines, Hoonah-Angoon, Juneau, Sitka, Wrangell-Petersburg, and Yakutat Counties in South East Alaska (2010)
- Letcher and Harlan Counties, Kentucky (2010)
- Pacific County, Washington and Clatsop County, Oregon (2011)
- National Survey with an oversample of non-metro counties (2011)

Questionnaire

The questionnaires used in the CERA surveys were developed by a team of researchers at the Carsey Institute at the University of New Hampshire with consultation with the UNH Survey Center. Questions used come largely from prior research studies. Questionnaires were pre-tested with a randomly sampled segment of the study population to determine interview length and to screen for questions that were difficult to answer. The final questionnaire was submitted and approved by the UNH Institutional Review Board (IRB). After the questionnaire had been finalized, it was programmed into Sawtooth Ci3 for interviewing.

How the Sample Was Selected

A sample of households in each of the CERA regions was selected by a procedure known as random digit dialing. The way this works is as follows. First, with the aid of the computer, an area code is selected at random from within the selected geography (e.g., 603). Next, one of the three-digit telephone exchanges which are currently used in the area code (e.g., 772) is randomly selected. The computer then randomly selects one of the "working blocks"-- the first two of the last four numbers in a telephone number (e.g., 64) -- and attaches it to the randomly selected exchange. Finally, the computer program then generates a two-digit random number between 00 and 99 (e.g., 57) which is attached to the previously selected prefix (772), and the previously selected working block (64) resulting in a complete telephone number -- i.e., 603/772-6457. This procedure is then repeated numerous times by the computer to generate more random numbers, so that we have a sufficient quantity to conduct the survey. The end result is that each household in the area in which there is a telephone has an equally likely chance of being selected into the sample.

The random samples used in the 2007, 2008, 2009 and 2010 CERA Surveys were purchased from Scientific Telephone Surveys (STS), of Fort Hill Ranch, California. STS screens each selected telephone number to eliminate non-working numbers, disconnected numbers, and business numbers to improve the efficiency of the sample, reducing the amount of time interviewers spend calling non-usable numbers.

Interviewing

Each of these randomly generated telephone numbers is called by an interviewer from either the centrally supervised facility at the UNH Survey Center or by one of three survey research companies that the Survey Center sub-contacted with: NSON research of Salt Lake City, Utah; Robinson and Muenster Associates of Sioux Falls, South Dakota; and Information Specialists Group (ISG) of Minneapolis, Minnesota. These research companies were used in order to complete the interviewing in the time desired frame and had partnered with the UNH Survey Center in prior research studies. In addition, these companies are located in the Central and Mountain time zones which improved productivity of

interviewing respondents in these regions of the country. Interviewers were trained and monitored by UNH Survey Center staff and daily call dispositions were received and monitored by UNH staff. All interviews were conducted using the same Sawtooth Ci3 computer assisted interviewing (CATI) software and all programming was done by UNH Survey Center staff.

If the number called is found not to be a residential one, it is discarded and another random number is called. (Approximately fifty percent of the numbers are discarded because they are found to be businesses, institutions, or not assigned.) If it is a residential number, the interviewer then randomly selects a member of the household by asking to speak with the adult currently living in the household who has had the most recent birthday. This selection process ensures that every adult (18 years of age or older) in the household has an equally likely chance of being included in the survey. No substitutions are allowed. If, for example, the randomly selected adult is not at home when the household is first contacted, the interviewer cannot substitute by selecting someone else who just happens to be there at the time. Instead, he or she must make an appointment to call back when the randomly selected adult is at home. In this way, respondent selection bias is minimized.

When the Interviewing Was Conducted

Respondents in the 2007, 2008, 2009 and 2010 CERA Surveys were interviewed during specified field periods in Table 1. Each selected respondent was called between 10:00 AM and 9:00 PM, local time.

Table 1 displays the field period for each region, the number of completed interviews, and the response rate. Response rates are calculated using the standard developed by the American Association for Public Opinion Research (AAPOR).[1] AAPOR response rate #4 was used because of the overlap of telephone exchanges across geographies in each of the regions. The formula to calculate AAPOR response rate #4 is:

$$\frac{I}{((I+P) + (R+NC+O) + e(UH+UO))}$$

I=Complete Interviews, **P**=Partial Interviews, **R**=Refusal and break off, **NC**=Non Contact, **O**=Other, **e**=estimated portion of cases of unknown eligibility that are eligible, **UH**=Unknown household, **UO**=Unknown other.

[1] For a discussion of the importance of understanding non-response and calculation of response rates, see Groves, Robert (2006) "Non-response Rates and Non-response Bias in Household Surveys," *Public Opinion Quarterly,* 70(5):646-675.

TABLE 1

Response Rates for the CERA Survey

Region	Year	Field Period	N Interviews	Response Rate
Alabama	2007	9/1/07 – 10/12/07	1010	19.2%
Colorado	2007	5/15/07 – 6/3/07	1006	22.5%
Kansas	2007	5/28/07 – 6/6/07	1008	25.7%
Kentucky	2007	5/18/07 – 6/4/07	1000	18.1%
Maine	2007	4/4/07 – 4/22/07	751	26.0%
Michigan	2008	8/1/08 – 8/19/08	1008	29.8%
Mississippi	2007	5/26/07 – 6/12/07	1000	23.0%
New Hampshire	2007	4/17/07-5/7/07, 7/27/07-8/8/07[2]	969	26.2%
Oregon / Washington	2007	5/17/07 – 6/11/07	1000	23.6%
Maine	2009	8/3/2010 – 9/8/2010	1526	40.0%
Maine	2010	6/6/2010 – 7/2/2010	754	28.2%
New Hampshire	2010	5/6/10-6/24/10, 6/12/10-6/15/10[3]	756	30.8%
Vermont	2010	5/14/2010 – 6/9/2010	354	35.4%
Louisiana	2010	7/29/2010 – 9/11/2010	1018	38.0%
Florida	2010	8/3/2010 – 9/30/2010	1034	41.0%
Alaska	2010	6/22/2010 – 8/2/2010	509	40.1%
Washington	2010	10/13/2010 – 11/15/2010	1020	36.5%
South East Alaska	2010	11/16/2010 – 12/21/2010	1022	31.9%
Kentucky	2010	11/12/2010 – 1/9/2011	1020	30.8%
Pacific Northwest	2011	1/14/2011 – 2/27/2011	1014	30.7%
National	2011	7/13/2011 – 8/21/2011	2006	31.1%

Weighting of the Data

To avoid biasing the sample in favor of households which can be reached through more than one telephone number, each case should be weighted inversely to its probability of being included in the sample. In addition, the data should be weighted to correct for sampling biases due to size of household (i.e., number of adults living in the household). Finally, the data should be weighted to correct for potential sampling biases on the sex of the respondent using, 2000 or 2010[4] U.S. Census figures.

---

[2] In 2007, in order to draw conclusions about the city of Berlin New Hampshire, which is undergoing particularly difficult economic stress, an oversample of 269 Berlin residents were interviewed between July 26 and August 8, 2007.

[3] There was an attempt to reach cell-only households in Coos County, however only 27 completed interviews were conducted via cell phone.

[4]Beginning with surveys conducted in 2011, 2010 census data were used for weighting.

Sampling Error

The CERA Surveys, like all surveys, are subject to sampling error due to the fact that all residents in the area were not interviewed. For those questions asked of one-thousand (1000) or so respondents, the error is +/-3.1%. For those questions where fewer than 1000 persons responded, the sampling error can be calculated as follows:

$$\text{Sampling error} = \pm (1.96) \sqrt{\frac{P(1-P)}{N}}$$

Where **P** is the percentage of responses in the answer category being evaluated and **N** is the total number of persons answering the particular question.

For example, suppose you had the following distribution of answers to the question, "Should the state spend more money on road repair even if that means higher taxes?" Assume 1,000 respondents answered the question as follows:

YES	- 47%
NO	- 48%
DON'T KNOW	- 5%

The sampling error for the "YES" percentage of 47% would be

$$\pm (1.96) \sqrt{\frac{(47)(53)}{1{,}000}} = \pm 3.1\%;$$

for the "NO" percentage of 48% it would be

$$\pm (1.96) \sqrt{\frac{(48)(52)}{1{,}000}} = \pm 3.1\%;$$

and for the "DON'T KNOW" percentage of 5% it would be

$$\pm (1.96) \sqrt{\frac{(5)(95)}{1{,}000}} = \pm 1.4\%;$$

In this case we would expect the true population figures to be within the following ranges:

YES	43.9% - 50.1% (i.e., 47% +/-3.1%)
NO	44.9% - 51.1% (i.e., 48% +/-3.1%)
DON'T KNOW	3.6% - 6.4% (i.e., 5% +/-1.4%)
