# Supplementary material for: Tracking Public Beliefs About Anthropogenic Climate Change
Source: PLoS One. 2015 Sep 30;10(9):e0138208. doi: 10.1371/journal.pone.0138208 (PMC4589389; doi:10.1371/journal.pone.0138208)
Supplement: S3 File — (PDF) [file pone.0138208.s003.pdf]

S3 File

Methodology Statement and Topline for iMediaEthics Poll on Climate Change

Conducted by Princeton Survey Research Associates International (PSRAI)

PSRAI obtained telephone interviews with a nationally representative sample of 1,002 adults living in the continental United States. Telephone interviews were conducted by landline (502) and cell phone (500, including 270 without a landline phone). Interviews were done in English and Spanish by Princeton Data Source from July 17-20, 2014. Statistical results are weighted to correct known demographic discrepancies. The margin of sampling error for the complete set of weighted data is ± 3.6 percentage points.

Final Topline Results
July 21, 2014

Total:	1,002 adults age 18 and older<br>Margin of error: plus or minus 3.6 percentage points
Half Sample A:	517 adults age 18 and older<br>Margin of error: plus or minus 5.0 percentage points
Half Sample B:	485 adults age 18 and older<br>Margin of error: plus or minus 5.1 percentage points
Interviewing dates:	July 17-20, 2014

LANDLINE INTRODUCTION:
Hello, my name is _________________ and I'm calling for Princeton Survey Research. We're conducting a study about some important issues today, and would like to include your household. [RANDOMIZE RESPONDENT SELECTION: May I please speak with the YOUNGEST ADULT MALE, age 18 or older, who is now at home? (IF NO MALE AT HOME NOW, ASK: May I please speak with the YOUNGEST ADULT FEMALE, age 18 or older, who is now at home?) / May I please speak with the YOUNGEST ADULT FEMALE, age 18 or older, who is now at home? (IF NO FEMALE AT HOME NOW, ASK: May I please speak with the YOUNGEST ADULT MALE, age 18 or older, who is now at home?)]

CELL PHONE INTRO:
Hello, I am ___ calling for Princeton Survey Research. We're conducting a study about some important issues today. [IF RESPONDENT SAYS DRIVING/UNABLE TO TAKE CALL: Thank you. We will try you another time...]

CELL PHONE SCREENING INTERVIEW:
S1. Are you under 18 years old, OR are you 18 or older? [CONTINUE IF 18 OR OLDER; ALL OTHERS TERMINATE]

READ TO ALL CELL PHONE – INTRODUCTION TO MAIN INTERVIEW: If you are now driving a car or doing any activity requiring your full attention, I need to call you back later. The first question is...

Notes: Due to rounding, percentages may not add to 100%. An asterisk (*) indicates values less than 0.5%. Volunteered responses are indicated by (VOL.).

1A Next, I would like to ask you some questions about the issue of global warming or climate change. How much do you feel you understand about this issue—would you say a great deal, a moderate amount, only a little, or nothing at all?

BASED ON HALF SAMPLE A

	%
A great deal	21
A moderate amount	41
Only a little	26
Nothing at all	11
(VOL.) Don't know	1
(VOL.) Refused	*

1B Next, I would like to ask you some questions about the issue of global warming or climate change. How closely are you following this issue: very closely, somewhat closely, not too closely, or not closely at all?

BASED ON HALF SAMPLE B

	%
Very closely	22
Somewhat closely	41
Not too closely	15
Not closely at all	21
(VOL.) Don't know	1
(VOL.) Refused	*

2 Which of the following statements comes closest to your opinion about climate change? [READ IN ORDER FOR SAMPLE C; READ IN REVERSE ORDER FOR SAMPLE D; ALWAYS READ 4 LAST]

	%
It is happening now, caused mainly by human activities	49
It is happening now, but caused mainly by natural forces	28
It is NOT happening now	7
Aren't sure about whether climate change is happening now	15
(VOL.) Refused	1

2A Do you feel pretty sure about that, or do you have a lot of doubt?

BASED ON HAS OPINION ON CLIMATE CHANGE (N=834)

	%
Feel pretty sure	87
Have a lot of doubt	11
(VOL.) Don’t know	2
(VOL.) Refused	0

2B Do you lean toward one view or another? [REPEAT 1-3 IF NECESSARY; ROTATE IN SAME ORDER AS UNH2]

BASED ON DOESN’T HAVE OPINION ON CLIMATE CHANGE (N=168)

	%
It is happening now, caused mainly by human activities	24
It is happening now, but caused mainly by natural forces	21
It is NOT happening now	10
(VOL.) Don’t lean/Still aren’t sure about whether climate change is happening now	39
(VOL.) Refused	7

3 Which of the following two statements do you think is more accurate? [READ IN ORDER FOR SAMPLE C; READ IN REVERSE ORDER FOR SAMPLE D; ALWAYS READ 3 LAST]

	%
Most scientists agree that climate change is happening now, caused mainly by human activities	44
There is little agreement among scientists whether climate change, caused mainly by human activities, is happening now	21
Unsure what scientists believe about climate change	33
(VOL.) Refused	3

3A Do you feel pretty sure about that, or do you have a lot of doubt?

BASED ON CHOSE A STATEMENT (N=662)

	%
Feel pretty sure	91
Have a lot of doubt	9
(VOL.) Don’t know	*
(VOL.) Refused	*

3B Do you lean toward one view or the other? [REPEAT 1-2 IF NECESSARY; ROTATE IN SAME ORDER AS UNH3]

BASED ON DID NOT CHOOSE A STATEMENT (N=340)

	%
Most scientists agree that climate change is happening now, caused mainly by human activities	29
There is little agreement among scientists whether climate change, caused mainly by human activities, is happening now	30
(VOL.) Don't lean/ Still unsure what scientists believe about climate change	34
(VOL.) Refused	8

4A Would you approve or disapprove of the Federal Government requiring power plants to reduce greenhouse gases, even if it would mean higher utility bills for consumers, or are you unsure?

BASED ON HALF SAMPLE A

	%
Approve	36
Disapprove	32
Unsure/Don't Know	31
(VOL.) Refused	1

4AA If the Federal Government DOES NOT require companies to reduce greenhouse gases, would you be… [READ]

BASED ON APPROVE OF FED GOVT REQUIREMENTS (N=198)

	%
Very upset	23
Somewhat upset	43
Not too upset	18
Not upset at all	15
(VOL.) Don't know	1
(VOL.) Refused	*

4AB If the Federal Government DOES require companies to reduce greenhouse gases, would you be… [READ]

BASED ON DISAPPROVE OF FED GOVT REQUIREMENTS (N=162)

	%
Very upset	46
Somewhat upset	29
Not too upset	8
Not upset at all	12
(VOL.) Don't know	4
(VOL.) Refused	1

4B Would you approve or disapprove of the Obama Administration requiring power plants to reduce greenhouse gases, even if it would mean higher utility bills for consumers, or are you unsure?

BASED ON HALF SAMPLE B

	%
Approve	42
Disapprove	28
Unsure/Don’t Know	30
(VOL.) Refused	*

4BA If the Obama Administration DOES NOT require companies to reduce greenhouse gases, would you be… [READ]

BASED ON APPROVE OF OBAMA ADMIN REQUIREMENTS (N=200)

	%
Very upset	29
Somewhat upset	41
Not too upset	16
Not upset at all	12
(VOL.) Don’t know	1
(VOL.) Refused	1

4BB If the Obama Administration DOES require companies to reduce greenhouse gases, would you be… [READ]

BASED ON DISAPPROVE OF OBAMA ADMIN REQUIREMENTS (N=143)

	%
Very upset	50
Somewhat upset	28
Not too upset	11
Not upset at all	9
(VOL.) Don’t know	1
(VOL.) Refused	0

DEMOGRAPHICS

I have one last set of questions to help us better understand the people who took part in our survey.

SEX Respondent’s sex [DO NOT ASK]

	%
Male	50
Female	50

EMPLOY Are you now employed full-time, part-time, or not employed?

	%
Employed full-time	46
Employed part-time	13
Not employed	40
(VOL.)Don't know/Refused	1

PAR Are you the parent or guardian of any children under 18 years of age?

	%
Yes	28
No	71
(VOL.) Don't know/Refused	1

AGE What is your age?

	%
18 to 29	21
30 to 49	32
50 to 64	27
65 and older	17
(VOL.) Refused	3

EDUC What is the highest level of school you have completed or the highest degree you have received? [DO NOT READ]

	%
Less than high school (Grades 1-8 or no formal schooling)	4
High school incomplete (Grades 9-11 or Grade 12 with NO diploma)	6
High school graduate (Grade 12 with diploma or GED certificate)	31
Some college, no degree (includes some community college)	18
Two year associate degree from a college/university	13
Four year college or university degree/Bachelor's degree	16
Some postgraduate or professional schooling, no postgraduate degree	2
Postgraduate or professional degree, including master's, doctorate, medical or law degree	10
Don't know/Refused	*

SUMMARY: RACE/ETHNICITY

HISP Are you of Hispanic or Latino origin, such as Mexican, Puerto Rican, Cuban or some other Spanish background?

RACE What is your race? Are you white, black, Asian, or some other race? [IF RESPONDENT SAYS HISPANIC OR LATINO, PROBE: Do you consider yourself a WHITE (Hispanic/Latino) or a BLACK (Hispanic/Latino)?

	%	
White, non-Hispanic	65	
Total non-White	33	
Black, non-Hispanic		12
Hispanic		15
Asian/Pacific Islander, non-Hispanic		2

Other, non-Hispanic	4
(VOL.) Don’t know/Refused	2

SUMMARY: INCOME

INC Last year – that is, in 2013 – approximately what was your total family income before taxes? Just tell me when I get to the right category.

INC1 [IF “DON’T KNOW” OR “REFUSED”, ASK:] Keeping in mind that this is a completely confidential survey, can you please tell me if your total household income BEFORE taxes last year was over or under $75,000?

INC2 [IF UNDER $75,000, ASK:] Was it over or under $50,000?

INC3 [IF UNDER $50,000, ASK:] Was it over or under $30,000?

	%
$75,000 or more	26
$50,000 to under $75,000	13
$30,000 to under $50,000	18
Under $30,000	31
Undesignated	11

REG Which of these statements best describes you? [READ IN ORDER]

	%
Are you ABSOLUTELY CERTAIN that you are registered to vote at your current address	74
Are you PROBABLY registered, but there is a chance your registration has lapsed, OR	3
Are you NOT registered to vote at your current address?	22
(VOL.) Don’t know/Refused	1

PARTY In politics TODAY, do you consider yourself a Republican, Democrat, or Independent?

	%
Republican	24
Democrat	30
Independent	32
(VOL.) No preference	8
(VOL.) Other party	1
(VOL.) Don't know/Refused	5

PARTYLN (If not “Republican or Democrat”) Do you lean toward one party or the other?

NET RESULT OF PARTY AND PARTYLN:

	%
Republican	24
Lean Republican	13
No lean-neither party	20
Lean Democrat	13
Democrat	30

That completes the interview. Thank you very much for your time and cooperation. Have a nice

day/evening.
