# Supplementary material for: Tracking Public Beliefs About Anthropogenic Climate Change
Source: PLoS One. 2015 Sep 30;10(9):e0138208. doi: 10.1371/journal.pone.0138208 (PMC4589389; doi:10.1371/journal.pone.0138208)
Supplement: S4 File — (PDF) [file pone.0138208.s004.pdf]

S4 File

Stata graphing commands and statistical tables for Hamilton, Hartter and Safford (2015) “Tracking public beliefs about anthropogenic climate change.” *PLOS One*.

Figure 1A

```
use S3_Dataset.dta
catplot warmop2_r [aw=tempwt] if type!=4, over(type) ///
       percent(type) asyvars l1title("") ///
              legend(position(5) ring(0) col(2) size(small) rowgap(*.01) ///
                     symxsize(*.8) symysize(*.7) order(1 3 2 4)) ///
       blabel(bar, format(%2.0f)) ytitle("") ///
       ylabel(0(10)60, grid gmin gmax) ///
       title("{bf:(A)}  US and New Hampshire surveys", size(medsmall) position(11))
```

Figure 1B

```
use S3_Dataset.dta
graph hbar (mean) warmop2d00 [aw=tempwt] if type==4, ///
       over(survname, sort(warmop2d00)) ytitle("") ///
       blabel(bar, format(%2.0f)) ylabel(0(10)60, grid gmin gmax) ///
       title("{bf:(B)}  Regional CERA/CAFOR surveys", size(medsmall) position(11)) ///
       bar(1, color(navy))
```

Figure 2A

```
use S4_Dataset.dta
graph twoway rarea warmophi warmoplo edate5 if survey<50, color(gs12) ///
       || connect warmop00 edate5 if survey<50, msymbol(d) lwidth(medium) ///
             mlwidth(medium) lcolor(dkorange) mcolor(dkorange) ///
       || rcap warmophi warmoplo edate5 if survey==80, lcolor(black) ///
             lwidth(medthick) ///
       || scatter warmop00 edate5 if survey==80, mcolor(black) msymbol(T) ///
             lwidth(medthick) mfcolor(white) ///
       || rcap warmophi warmoplo edate5 if survey==90, lcolor(black) lwidth(medthick) ///
       || scatter warmop00 edate5 if survey==90, mcolor(black) msymbol(T) ///
       || rcap warmophi warmoplo edate5 if survey==100, lcolor(black) lwidth(medthick) ///
       || scatter warmop00 edate5 if survey==100, mcolor(black) msymbol(S) ///
             mfcolor(white) lwidth(medthick) ///
       || , xtitle("") legend(order(2 1 4 6 8) col(3) position(5) ring(0) ///
             label(2 "New Hampshire") label(1 "95% conf. interval") ///
             label(4 "US NCERA") label(6 "US GSS") label(8 "US IME") ///
             size(small) rowgap(*.01) holes(3)) ///
       ytick(0(10)100, grid gmax gmin) ylabel(0(20)100) ///
       ytitle("") xlabel(, labsize(small)) ///
       xlabel(18374 18466 18531 18658 18735 18803 18910 19021 19098 19184 ///
             19269 19391 19454 19555 19635 19753 19814 19913 20005 20128 ///
             20225 , labsize(small) angle(30)) ///
       xscale(range(18370 20230)) text(97 18380 "{bf:(A)}")
```

Figure 2B

```
use C:\trip\A_research\Granite_State_Poll\Synthesis\Supporting_information\S5_Dataset.dta, clear
graph twoway rarea warmophi warmoplo edate5 if party3==1 & survey<70, color(gs14) ///
        || rarea warmophi warmoplo edate5 if party3==3 & survey<70, color(gs14) ///
        || rarea warmophi warmoplo edate5 if party3==2 & survey<70, color(gs12) ///
                legend(position(5) ring(0) ) ///
        || connect warmop00 edate5 if party3==1 & survey<70, msymbol(O) lwidth(medium) ///
                mlwidth(medium) lcolor(navy) mcolor(navy) ///
        || line warmop00 edate5 if party==2 & survey<70, lwidth(medium) lcolor(dkgreen) ///
        || connect warmop00 edate5 if party==3 & survey<70, msymbol(S) lwidth(medium) ///
                mlwidth(medium) clpattern(longdash) lcolor(maroon) mcolor(maroon) ///
        || rcap warmophi warmoplo edate5 if party3==1 & survey==80, lcolor(black) lwidth(medthick) ///
        || rcap warmophi warmoplo edate5 if party3==2 & survey==80, lcolor(black) lwidth(medthick) ///
        || rcap warmophi warmoplo edate5 if party3==3 & survey==80, lcolor(black) lwidth(medthick) ///
        || scatter warmop00 edate5 if party3==1 & survey==80, mcolor(black) msymbol(T) ///
                lwidth(medthick) mfcolor(white) ///
        || scatter warmop00 edate5 if party3==2 & survey==80, mcolor(black) msymbol(T) ///
                lwidth(medthick) mfcolor(white) ///
        || scatter warmop00 edate5 if party3==3 & survey==80, mcolor(black) msymbol(T) ///
                lwidth(medthick) mfcolor(white) ///
        || rcap warmophi warmoplo edate5 if party3==1 & survey==90, lcolor(black) lwidth(medthick) ///
        || rcap warmophi warmoplo edate5 if party3==2 & survey==90, lcolor(black) lwidth(medthick) ///
        || rcap warmophi warmoplo edate5 if party3==3 & survey==90, lcolor(black) lwidth(medthick) ///
        || scatter warmop00 edate5 if party3==1 & survey==90, mcolor(black) msymbol(T) ///
        || scatter warmop00 edate5 if party3==2 & survey==90, mcolor(black) msymbol(T) ///
        || scatter warmop00 edate5 if party3==3 & survey==90, mcolor(black) msymbol(T) ///
        || rcap warmophi warmoplo edate5 if party3==1 & survey==100, lcolor(black) lwidth(medthick) ///
        || rcap warmophi warmoplo edate5 if party3==2 & survey==100, lcolor(black) lwidth(medthick) ///
        || rcap warmophi warmoplo edate5 if party3==3 & survey==100, lcolor(black) lwidth(medthick) ///
        || scatter warmop00 edate5 if party3==1 & survey==100, mcolor(black) msymbol(S) mfcolor(white) ///
        || scatter warmop00 edate5 if party3==2 & survey==100, mcolor(black) msymbol(S) mfcolor(white) ///
        || scatter warmop00 edate5 if party3==3 & survey==100, mcolor(black) msymbol(S) mfcolor(white) ///
        || , xtitle("") ///
        legend(order(4 3 10 5 16 6 22) col(3) row(3) holes(5 8) position(5) ring(0) ///
                label(4 "NH Democrat") label(5 "NH Independent") ///
                label(6 "NH Republican") label(10 "US NCERA") label(16 "US GSS") label(22 "US IME") ///
                label(3 "95% c.i.") rowgap(*.01) symxsize(*.7) size(small) bmargin(zero)) ///
        ytick(0(10)100, grid gmax gmin) ylabel(0(20)100) ytitle("") ///
        xlabel(18374 18466 18531 18658 18735 18803 18910 19021 19098 19184 ///
                19269 19391 19454 19555 19635 19753 19814 19913 20005 20128 ///
                20225 , labsize(small) angle(30)) ///
        xscale(range(18370 20230)) text(97 18380 "{bf:(B)}")
```

Table 2

General Social Survey (GSS) data can be downloaded freely from the National Opinion Research Center (NORC) website at http://www3.norc.org/GSS+Website/

Citation for using these data is:
Smith, Tom W, Peter Marsden, Michael Hout, and Jibum Kim. General Social Surveys, 1972-2012 [machine-readable data file] /Principal Investigator, Tom W. Smith; Co-Principal Investigator, Peter V. Marsden; Co-Principal Investigator, Michael Hout; Sponsored by National Science Foundation. --NORC ed.-- Chicago: National Opinion Research Center [producer]; Storrs, CT: The Roper Center for Public Opinion Research, University of Connecticut [distributor], 2013.
1 data file (57,061 logical records) + 1 codebook (3,432p.). -- (National Data Program for the Social Sciences, No. 21).

For our analysis we used the 2012 GSS panel survey, which includes the climate beliefs question as a variable named *clmtchng*. This variable and some others in the dataset were renamed or recoded for consistency with the other datasets used in our paper. Under the General Social Survey terms of use (http://www.icpsr.umich.edu/icpsrweb/ICPSR/studies/35478/terms) we are not authorized to publish the GSS subset, but interested researchers can construct their own version with the same properties.

```
. use S7_Dataset.dta
(2012 General Social Survey (Smith 2013) warming subset)

. svy: logit warmop2d age3 sex2 c.educ40##c.party30 , or nolog
(running logit on estimation sample)

Survey: Logistic regression

Number of strata   =         1                  Number of obs      =      1,242
Number of PSUs     =     1,242                  Population size    = 1,163.0448
                                                Design df          =      1,241
                                                F(   5,    1237)   =      12.90
                                                Prob > F           =     0.0000

------------------------------------------------------------------------------
             |             Linearized
    warmop2d | Odds Ratio   Std. Err.      t    P>|t|     [95% Conf. Interval]
-------------+----------------------------------------------------------------
        age3 |   .9946053   .0043548    -1.24   0.217     .9860984    1.003186
        sex2 |   1.116041   .1593299     0.77   0.442     .8434144    1.476792
      educ40 |   1.209783   .0824681     2.79   0.005     1.058343    1.382893
     party30 |    .565585   .0452725    -7.12   0.000     .4833888     .661758
             |
    c.educ40#|
   c.party30 |    .820975   .0603328    -2.68   0.007     .7107464    .9482987
             |
       _cons |   1.406893   .3521503     1.36   0.173     .8609823    2.298941
------------------------------------------------------------------------------
```

```
. use S1_Dataset.dta
(National CERA survey, August 2011)

. svy: logit warmop2d age3 sex2 c.educ40##c.party30 , or nolog
(running logit on estimation sample)

Survey: Logistic regression

Number of strata   =         2                  Number of obs     =      1,714
Number of PSUs     =     1,714                  Population size   = 1,686.2411
                                                Design df         =      1,712
                                                F(   5,   1708)   =      29.25
                                                Prob > F          =     0.0000

------------------------------------------------------------------------------
             |             Linearized
    warmop2d | Odds Ratio   Std. Err.      t    P>|t|     [95% Conf. Interval]
-------------+----------------------------------------------------------------
        age3 |   .9870372   .0040313    -3.19   0.001      .979162    .9949757
        sex2 |   1.129339   .1647735     0.83   0.405     .8482893    1.503505
      educ40 |   1.248718   .0868162     3.19   0.001     1.089541    1.431151
     party30 |   .4822449   .0418298    -8.41   0.000     .4068012    .5716799
             |
    c.educ40#|
   c.party30 |   .7851328   .0601261    -3.16   0.002     .6756338    .9123783
             |
       _cons |   1.548629   .3840997     1.76   0.078     .9520906    2.518933
------------------------------------------------------------------------------


. use S2_Dataset.dta
(Climate poll 7/2014: Art Science Research Laboratory's iMediaEthics news site)

. svy: logit warmop2d age3 sex2 c.educ40##c.party30 , or nolog
(running logit on estimation sample)

Survey: Logistic regression

Number of strata   =         1                  Number of obs     =        960
Number of PSUs     =       960                  Population size   =  973.78931
                                                Design df         =        959
                                                F(   5,    955)   =      22.50
                                                Prob > F          =     0.0000

------------------------------------------------------------------------------
             |             Linearized
    warmop2d | Odds Ratio   Std. Err.      t    P>|t|     [95% Conf. Interval]
-------------+----------------------------------------------------------------
        age3 |   .9846979   .0043069    -3.53   0.000      .976282    .9931863
        sex2 |   1.073713    .179476     0.43   0.671     .7734397    1.490563
      educ40 |   1.218158   .0913055     2.63   0.009     1.051531    1.411188
     party30 |   .4272597   .0390873    -9.30   0.000     .3570447     .511283
             |
    c.educ40#|
   c.party30 |   .8502985   .0717101    -1.92   0.055     .7206004    1.003341
             |
       _cons |   2.246296   .5249632     3.46   0.001         1.42    3.553412
------------------------------------------------------------------------------
```

(Under terms of human-subjects agreements the CERA/CAFOR survey microdata are not published; contact lead author with questions.)

```
. svy: logit warmop2d age sex c.educ40##c.party30 i.survfips, nolog or
(running logit on estimation sample)

Survey: Logistic regression

Number of strata   =        38                  Number of obs     =     11,554
Number of PSUs     =    11,554                  Population size   = 11,268.549
                                                Design df         =     11,516
                                                F(  42,  11475)   =      26.69
                                                Prob > F          =     0.0000

------------------------------------------------------------------------------
             |             Linearized
    warmop2d | Odds Ratio   Std. Err.      t    P>|t|     [95% Conf. Interval]
-------------+----------------------------------------------------------------
         age |   .9826188   .0017683    -9.74   0.000     .9791588     .986091
         sex |   1.213116   .0689782     3.40   0.001      1.08517    1.356148
      educ40 |   1.205133   .0339444     6.62   0.000       1.1404    1.273541
     party30 |   .4246923   .0143653   -25.32   0.000      .397447    .4538053
             |
    c.educ40#|
   c.party30 |   .7477278   .0233659    -9.30   0.000     .7033012    .7949607
             |
    survfips |
     121133  |   1.332222    .248508     1.54   0.124     .9242296     1.92032
     241007  |   1.547137   .2644644     2.55   0.011     1.106651    2.162951
     253049  |    2.31303   .4837032     4.01   0.000     1.535177    3.485011
     412005  |   1.446815   .2713868     1.97   0.049     1.001686    2.089749
     412037  |   1.133689   .2769923     0.51   0.608     .7022628    1.830158
     412045  |   .9999195   .2505839    -0.00   1.000     .6118261    1.634188
     522075  |   1.577306   .2911428     2.47   0.014     1.098456    2.264901
     522109  |   1.188552   .2297199     0.89   0.371     .8137351    1.736014
     602130  |   1.274279   .2713728     1.14   0.255     .8394059    1.934448
     602198  |   2.186882   .6754482     2.53   0.011     1.193693    4.006436
     723017  |   2.723553   .4561375     5.98   0.000     1.961384    3.781892
     733007  |    2.53698   .4285731     5.51   0.000     1.821831    3.532856
     750001  |   3.091551   .6237871     5.59   0.000     2.081661    4.591374
     841001  |   1.422353   .2592068     1.93   0.053     .9951072    2.033036
     841061  |   1.136534   .2064427     0.70   0.481     .7960709    1.622605
     841063  |   1.121922   .2187857     0.59   0.555      .765513    1.644268
     953009  |   1.716941   .3159119     2.94   0.003     1.197072     2.46258
     953027  |   1.801613   .3356905     3.16   0.002     1.250381    2.595856
    1053033  |   2.407931   .5022291     4.21   0.000     1.599882    3.624101
    1053035  |   1.649585    .377941     2.18   0.029     1.052769    2.584737
    1053045  |   2.222048   .4148864     4.28   0.000     1.541011    3.204062
    1053053  |   1.948272   .4658791     2.79   0.005     1.219229     3.11325
    1102100  |   2.039451   .5595467     2.60   0.009      1.19111    3.492004
    1102105  |      2.439   1.115743     1.95   0.051     .9949039    5.979193
    1102110  |   1.907149   .3576145     3.44   0.001     1.320556    2.754308
    1102195  |   2.185593   .6661579     2.57   0.010     1.202541    3.972269
    1102220  |   1.793717   .4649889     2.25   0.024     1.079125    2.981507
    1102230  |   1.393854   1.007352     0.46   0.646     .3380499    5.747168
    1102275  |   1.389508   .6273221     0.73   0.466     .5734931    3.366619
    1102282  |   2.311761   1.215342     1.59   0.111     .8248991    6.478656
    1341001  |   2.421155   .5215431     4.10   0.000      1.58725    3.693176
    1341013  |   2.534387   .5702705     4.13   0.000     1.630501     3.93935
    1341023  |   1.985764   .4557868     2.99   0.003      1.26629    3.114024
    1341059  |   2.568627   .5302384     4.57   0.000     1.713839    3.849745
    1341061  |   1.367164   .3609072     1.18   0.236     .8148818    2.293752
    1341063  |   1.821769   .4347292     2.51   0.012     1.141168    2.908286
    1341069  |    1.81558    .523943     2.07   0.039     1.031213    3.196556
             |
       _cons |   1.057132   .1729142     0.34   0.734      .767157    1.456714
------------------------------------------------------------------------------
```

```
. test 121095.survfips 121133.survfips 241007.survfips 253049.survfips 412005.survfips ///
       412037.survfips 412045.survfips 522075.survfips 522109.survfips 602130.survfips ///
       602198.survfips 723017.survfips 733007.survfips 750001.survfips 841001.survfips ///
       841061.survfips 841063.survfips 953009.survfips 953027.survfips 1053033.survfips ///
       1053035.survfips 1053045.survfips 1053053.survfips 1102100.survfips 1102105.survfips ///
       1102110.survfips 1102195.survfips 1102230.survfips 1102275.survfips 1102282.survfips ///
       1341001.survfips 1341013.survfips 1341023.survfips 1341059.survfips 1341061.survfips ///
       1341063.survfips 1341069.survfips

Adjusted Wald test

 ( 1)  [warmop2d]121095b.survfips = 0
 ( 2)  [warmop2d]121133.survfips = 0
 ( 3)  [warmop2d]241007.survfips = 0
 ( 4)  [warmop2d]253049.survfips = 0
 ( 5)  [warmop2d]412005.survfips = 0
 ( 6)  [warmop2d]412037.survfips = 0
 ( 7)  [warmop2d]412045.survfips = 0
 ( 8)  [warmop2d]522075.survfips = 0
 ( 9)  [warmop2d]522109.survfips = 0
 (10)  [warmop2d]602130.survfips = 0
 (11)  [warmop2d]602198.survfips = 0
 (12)  [warmop2d]723017.survfips = 0
 (13)  [warmop2d]733007.survfips = 0
 (14)  [warmop2d]750001.survfips = 0
 (15)  [warmop2d]841001.survfips = 0
 (16)  [warmop2d]841061.survfips = 0
 (17)  [warmop2d]841063.survfips = 0
 (18)  [warmop2d]953009.survfips = 0
 (19)  [warmop2d]953027.survfips = 0
 (20)  [warmop2d]1053033.survfips = 0
 (21)  [warmop2d]1053035.survfips = 0
 (22)  [warmop2d]1053045.survfips = 0
 (23)  [warmop2d]1053053.survfips = 0
 (24)  [warmop2d]1102100.survfips = 0
 (25)  [warmop2d]1102105.survfips = 0
 (26)  [warmop2d]1102110.survfips = 0
 (27)  [warmop2d]1102195.survfips = 0
 (28)  [warmop2d]1102230.survfips = 0
 (29)  [warmop2d]1102275.survfips = 0
 (30)  [warmop2d]1102282.survfips = 0
 (31)  [warmop2d]1341001.survfips = 0
 (32)  [warmop2d]1341013.survfips = 0
 (33)  [warmop2d]1341023.survfips = 0
 (34)  [warmop2d]1341059.survfips = 0
 (35)  [warmop2d]1341061.survfips = 0
 (36)  [warmop2d]1341063.survfips = 0
 (37)  [warmop2d]1341069.survfips = 0
       Constraint 1 dropped

       F( 36, 11481) =    4.15
            Prob > F =    0.0000
```

(Under terms of human-subjects agreements the GSP survey microdata are not published; contact Lawrence.Hamilton@unh.edu with questions.)

```
. svy: logit warmop2d age3 sex2 c.educ40##c.party30 anomnh ib1.season year, nolog or
(running logit on estimation sample)

Survey: Logistic regression
```

```
Number of strata   =         1                 Number of obs     =     10,567
Number of PSUs     =    10,567                 Population size   = 10,521.322
                                               Design df         =     10,566
                                               F(  10,  10557)   =     144.81
                                               Prob > F          =     0.0000

------------------------------------------------------------------------------
             |             Linearized
    warmop2d | Odds Ratio   Std. Err.      t    P>|t|     [95% Conf. Interval]
-------------+----------------------------------------------------------------
        age3 |   .9853482   .0016511    -8.81   0.000     .9821171    .9885899
        sex2 |   1.334407   .0703616     5.47   0.000     1.203373    1.479708
      educ40 |   1.202421   .0307697     7.20   0.000     1.143594    1.264274
     party30 |   .3890908   .0131018   -28.03   0.000     .3642381    .4156393
             |
    c.educ40#|
   c.party30 |   .7803595   .0228061    -8.49   0.000     .7369116     .826369
             |
      anomnh |   1.017645   .0070089     2.54   0.011     1.003999    1.031477
             |
      season |
     Spring  |   1.025691    .074374     0.35   0.726     .8897912    1.182348
     Summer  |   1.144782   .0913674     1.69   0.090      .978991    1.338649
       Fall  |   1.027548   .0830864     0.34   0.737     .8769341     1.20403
             |
        year |   1.066859   .0186427     3.70   0.000     1.030934    1.104035
       _cons |   5.64e-57   1.98e-55    -3.68   0.000     6.47e-87    4.92e-27
------------------------------------------------------------------------------
```

Figure 3A

```
use  S7_Dataset.dta
svy: logit warmop2d age3 sex2 c.educ40##c.party30 , or nolog
margins, at(educ40 = (-1(1)2) party30 = (-1(1)1))
marginsplot, legend(position(11) ring(0) col(1) size(small) rowgap(*.01) ///
       symxsize(*.4)) ///
       title("{bf:(A)}  GSS national survey", ///
              position(11) size(medsmall)) xtitle("") ytitle("") ///
       plot1opts(lpattern(longdash) lwidth(medium) msymbol(O) ///
              mlwidth(medium)) ///
       plot2opts(lpattern(solid) lwidth(medium) msymbol(i) ///
              mlwidth(medium) lcolor(dkgreen) mcolor(dkgreen)) ///
       plot3opts(lpattern(dash) lwidth(medium) msymbol(Sh) ///
              mlwidth(medium) lcolor(maroon) mcolor(maroon)) ///
       xlabel(-1 "<= HS" 0 "Some coll" 1 "College" 2 "Postgrad") ///
       xscale(range(-1.1 2.1)) recastci(rarea) ci1opts(color(gs14)) ///
       ci2opts(color(gs13)) ci3opts(color(gs14)) ylabel(.2(.1).9, grid gmin gmax)
```

Figure 3B

```
use S1_Dataset.dta
svy: logit warmop2d age3 sex2 c.educ40##c.party30 , or nolog
margins, at(educ40 = (-1(1)2) party30 = (-1(1)1))
marginsplot, legend(position(11) ring(0) col(1) size(small) rowgap(*.01)
symxsize(*.4)) ///
       title("{bf:(B)}  NCERA national survey", ///
              position(11) size(medsmall)) xtitle("") ytitle("") ///
       plot1opts(lpattern(longdash) lwidth(medium) msymbol(O) ///
              mlwidth(medium)) ///
       plot2opts(lpattern(solid) lwidth(medium) msymbol(i) ///
              mlwidth(medium) lcolor(dkgreen) mcolor(dkgreen)) ///
       plot3opts(lpattern(dash) lwidth(medium) msymbol(Sh) ///
              mlwidth(medium) lcolor(maroon) mcolor(maroon)) ///
```

```
        xlabel(-1 "<= HS" 0 "Some coll" 1 "College" 2 "Postgrad") ///
        xscale(range(-1.1 2.1)) recastci(rarea) ci1opts(color(gs14)) ///
        ci2opts(color(gs13)) ci3opts(color(gs14)) ylabel(.2(.1).9, grid gmin gmax)
```

Figure 3C

(Under terms of human-subjects agreements the CERA/CAFOR survey microdata are not published; contact Lawrence.Hamilton@unh.edu with questions.)

```
use "C:\data\CERA_CAFOR_warmop.dta", clear
svy: logit warmop2d age sex c.educ40##c.party30, or nolog
label values party30 partyfoo
label define partyfoo -1 Dem 0 Ind 1 Rep
margins, at(educ40 = (-1(1)2) party30 = (-1(1)1))
marginsplot, legend(position(11) ring(0) col(1) size(small) rowgap(*.01) ///
                symxsize(*.4)) ///
        title("{bf:(C)}  CERA/CAFOR 11 regional surveys", ///
                position(11) size(medsmall)) xtitle("") ytitle("") ///
        plot1opts(lpattern(longdash) lwidth(medium) msymbol(O) ///
                mlwidth(medium)) ///
        plot2opts(lpattern(solid) lwidth(medium) msymbol(i) ///
                mlwidth(medium) lcolor(dkgreen) mcolor(dkgreen)) ///
        plot3opts(lpattern(dash) lwidth(medium) msymbol(Sh) ///
                mlwidth(medium) lcolor(maroon) mcolor(maroon)) ///
        xlabel(-1 "<= HS" 0 "Some coll" 1 "College" 2 "Postgrad") ///
        xscale(range(-1.1 2.1)) recastci(rarea) ci1opts(color(gs14)) ///
        ci2opts(color(gs13)) ci3opts(color(gs14)) ylabel(.2(.1).9, grid gmin gmax)
```

Figure 3D

(Under terms of human-subjects agreements the GSP survey microdata are not published; contact Lawrence.Hamilton@unh.edu with questions.)

```
svy: logit warmop2d age3 sex2 c.educ40##c.party30 anomnh year ib1.season, nolog or
quietly margins, at(educ40 = (-1(1)2) party30 = (-1(1)1))
marginsplot, legend(position(11) ring(0) col(1) size(small) rowgap(*.01)
symxsize(*.4)) ///
        title("{bf:(D)}  GSP New Hampshire 21 surveys", ///
                        position(11) size(medsmall)) xtitle("") ytitle("") ///
        plot1opts(lpattern(longdash) lwidth(medium) msymbol(O) ///
                mlwidth(medium)) ///
        plot2opts(lpattern(solid) lwidth(medium) msymbol(i) ///
                mlwidth(medium) lcolor(dkgreen) mcolor(dkgreen)) ///
        plot3opts(lpattern(dash) lwidth(medium) msymbol(Sh) ///
                mlwidth(medium) lcolor(maroon) mcolor(maroon)) ///
        xlabel(-1 "<= HS" 0 "Some coll" 1 "College" 2 "Postgrad") ///
        xscale(range(-1.1 2.1)) recastci(rarea) ci1opts(color(gs14)) ///
        ci2opts(color(gs13)) ci3opts(color(gs14)) ylabel(.2(.1).9, grid gmin gmax)
```
